# Microgravity effects on secondary metabolism of plant-affecting bacteria

**DOI:** 10.1128/spectrum.02239-25

**Published:** 2026-03-30

**Authors:** Gayatri Sharma, Mohammed M. A. Ahmed, Tahir Ali, Paul D. Boudreau, Patrick D. Curtis

**Affiliations:** 1 Department of Biology, University of Mississippi 251817, University, Mississippi, USA; 2 Department of BioMolecular Sciences, University of Mississippi 551786, University, Mississippi, USA; Universitat Wien, Vienna, Austria

**Keywords:** simulated microgravity, biosynthetic gene clusters, transcriptomics, targeted metabolomics, genetic engineering, secondary metabolites, NASA, clinostat

## Abstract

Despite their miniscule mass, microgravity has been shown to impose physiological stresses on bacteria, leading to altered gene regulation and metabolic output in unpredictable ways. Changes in metabolic output can ultimately impact the production of bacterial secondary metabolites. While a few studies have explored the changes in bacterial secondary metabolites under simulated or spaceflight microgravity, the specific area of plant-beneficial compound production by bacteria remains largely unexplored in these contexts. In this study, the production of metabolites pyoluteorin, 2,4-diacetylphloroglucinol (DAPG), orfamide A produced by the plant-promoting bacterium *Pseudomonas protegens* Pf-5, and pyrroloquinoline quinone (PQQ) produced by *Burkholderia ambifaria* AMMD under simulated microgravity was analyzed. Transcriptomic analysis of the biosynthetic clusters, coupled with targeted metabolomics, revealed significant downregulation of *ofaABC* and *pqqBCDE* operons on Day 3 of incubation under simulated microgravity. This downregulation corresponded to reduced levels of orfamide A and pyrroloquinoline quinone, indicating a functional link between transcriptional suppression and decreased metabolite yield. Two complementary genetic engineering approaches were used to increase the production of these compounds: native chromosomal promoter replacement with either endogenous or inducible promoters, or plasmid-based operon overexpression. While the chromosomal strategy yielded modest expression improvements in *P. protegens*, the plasmid-based system enabled robust expression with a significant increase in pyrroloquinoline production in *B. ambifaria*. This study provides a first step for engineering these microbes to provide plant-promoting metabolites in microgravity as a mechanism of aiding the cultivation of plants aboard space-faring vehicles.

**IMPORTANCE:**

Bacteria play an essential role in supporting plant health by producing antifungal compounds that protect against fungal diseases. In space, these bacterial functions may be disrupted. This study examined how microgravity affects the production of such compounds in plant beneficial bacteria, *Pseudomonas protegens* and *Burkholderia ambifaria*. Under simulated microgravity, both bacteria showed reduced production of some antifungal compounds due to reduced gene expression. To address this, we used genetic strategies to boost the bacteria's ability to produce these compounds despite the stress of microgravity. Our results showed that engineered bacteria could either partially recover gene expression or even enhance production. These findings are important first steps for engineering microbes to not only enhance plant growth for space agriculture but also provide important information for engineering microbes to boost crop production on Earth as well.

## INTRODUCTION

Bacterial adaptation involves changes in physiological behavior triggered by environmental factors (1). Under spaceflight conditions, this adaptation becomes particularly critical, as individual bacteria exhibit unique responses to the low-shear environment, leading to altered genetics and molecular behaviors (2–5). Despite growing evidence of these altered responses, many aspects of microbial behavior in microgravity remain poorly understood. As space exploration expands and missions lengthen, exposure to diverse and potentially unpredictable microbial communities becomes increasingly inevitable, making it critically important to understand their interactions with biological systems. This is especially relevant for plant cultivation, which is emerging as a cornerstone of life-support systems in space by providing essential functions such as oxygen generation, food production, and waste recycling. However, because microbes inevitably interact with plants in this closed environment, understanding and managing those interactions is crucial, particularly for preventing and controlling plant diseases. Several studies have shown that plants become more susceptible to fungal *Fusarium oxysporum* infections in microgravity environments (6–8). Additionally, a diverse array of bacterial and fungal genera has been reported to colonize the leaf and root tissues of space-grown lettuce, a nutritionally dense leafy green frequently used as a model in space-based agriculture (9–11). Given the confined yet microbiologically diverse environment of space habitats, effective disease management is essential to maintain biological system health without relying on external chemical inputs, as their application is not feasible in the closed environment of space-faring vehicles due to safety concerns (12, 13). One promising strategy to strengthen plant health involves the use of beneficial plant-associated bacteria with biocontrol potential (14). They can enhance plant defense responses and inhibit the growth of pathogenic contaminants (15). Such biological approaches may be crucial for ensuring food security during long-duration missions, minimizing payload demands, and avoiding chemically mediated disruptions in an already unpredictable environment (8).

Bacteria are known to impact plants through multiple mechanisms, such as nitrogen fixation, nutrient solubilization, phytohormone synthesis, synthesis of lytic enzymes, and the production of specialized metabolites, including siderophores and biosurfactants, that can suppress pathogens or modulate plant responses (15). Since bacteria regulate the synthesis of secondary metabolites in response to environmental cues (16), it is not surprising that several microgravity-based studies have reported altered production of these compounds under spaceflight conditions (3, 17–20). Although not essential for immediate survival, secondary metabolites support critical bacterial behaviors such as cell-cell signaling (21), microbial competition (22), and environmental adaptation (16). Some of these metabolites are known to influence plant growth (23, 24), which is especially relevant for space-based agriculture. For instance, *Bacillus subtilis* UD1022, a well-characterized plant growth-promoting rhizobacterium, is known terrestrially for promoting stomatal closure and limiting the entry of *Salmonella enterica* (25). However, under simulated microgravity, UD1022 lost its ability to restrict stomatal opening in lettuce, significantly reducing its effectiveness against *S. enterica* (26). Recent studies have shown that microbial volatile organic compounds (VOCs), a class of secondary metabolites, can regulate plant responses by inducing reactive oxygen species (ROS) signaling, which modulates stomatal closure and enhances stress resilience. For example, VOCs such as acetoin, produced by *Bacillus amyloliquefaciens* FZB42, were found to trigger ROS accumulation and influence both stomatal aperture and root architecture in *Arabidopsis* in a jasmonic acid-dependent manner (27). These findings suggest that stomatal regulation can also be mediated through bacterial secondary metabolites like VOCs. In light of this, the reduced effectiveness of *B. subtilis* UD1022 under microgravity may reflect not only disrupted bacterial-plant signaling but also impaired production of key secondary metabolites. This further raises the possibility that microgravity conditions interfere with essential metabolic pathways in biocontrol agents, potentially compromising the synthesis of compounds vital for plant growth.

Different bacterial species respond uniquely to microgravity, and their secondary metabolic profiles may vary significantly, adding another layer of complexity to microbial risk and utility assessments in space (20). As space agriculture advances, interest in biocontrol strains and their natural products has grown. However, since no single metabolite or mechanism is universally effective, understanding how secondary metabolites are regulated under microgravity and how they impact plant growth remains a critical gap. Addressing this is key to leveraging plant growth promoters for plant resilience and developing sustainable protection strategies in space. To address these knowledge gaps, this study utilized well-characterized plant-beneficial bacteria *Pseudomonas protegens* and *Burkholderia ambifaria. P. protegens* is known for its ability to suppress plant diseases through the production of diverse antimicrobial secondary metabolites such as pyrrolnitrin, pyoluteorin, 2,4-diacetylphloroglucinol (DAPG), orfamide A, and toxoflavin (28). These compounds often act synergistically to inhibit a broad range of plant pathogens (29, 30), enhancing *P. protegens* biocontrol potential and making it an ideal candidate for microgravity studies. Notably, orfamide-type cyclic lipopeptides (CLPs), produced by *Pseudomonas* species, have been linked to the lysis of oomycete zoospores and suppression of *Rhizoctonia*, a common soil-borne fungal pathogen (29). Additionally, compounds such as pyrrolnitrin and DAPG have been shown to reduce *Fusarium* spp. mycelial growth by 30%–40% (30), a fungus known to contaminate plants in space. Similarly, *Burkholderia* species produce potent antifungal compounds, including quinolinones, phenylpyrroles, and phenazines (31). Their effectiveness as biocontrol agents has led to the U.S. EPA registration of three strains for commercial use (32). Notably, *B. ambifaria* has shown strong activity against *Pythium* spp. and *Aphanomyces euteiches* fungal-like root pathogens (33). In this study, simulated microgravity was generated using a Rotary Cell Culture System: Clinostat, a ground-based platform that continuously reorients cultures to randomize the gravity vector, minimize sedimentation, and reduce the convective mixing normally caused by gravity. As a result, the cells remain in prolonged suspension with minimal mechanical agitation, creating a quiescent fluid environment dominated by diffusion rather than convection. While this setup does not fully replicate the microgravity experienced in orbit, it effectively models a low-shear, diffusion-limited environment. This system allows for the controlled investigation of microgravity-associated stresses, including altered nutrient diffusion, mechanical shear, and oxygen transfer, under laboratory conditions and has been widely used to study the effects of simulated weightlessness on bacterial cells (34–38). Understanding how such simulated microgravity conditions alter metabolite production and how these changes impact plant-bacterial interactions is essential for developing sustainable, microbe-assisted cultivation systems in space.

## RESULTS

### Simulated microgravity delays the onset of the exponential growth phase

**Fig 1:**
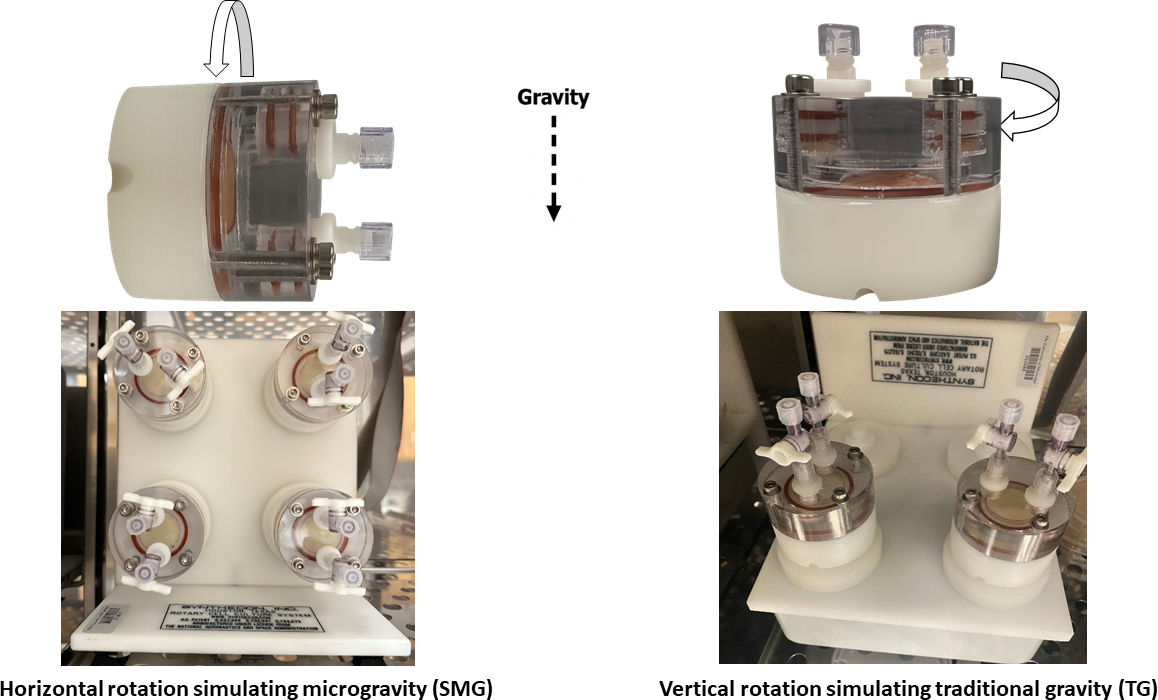
Ground-based hardware used to simulate microgravity on Earth. A rotatory cell culture system (RCCS) equipped with HARVs and clinostat vessels rotated at different rotational axes: horizontal rotation to simulate microgravity (left) and vertical rotation for traditional gravity (right).

**Fig 2:**
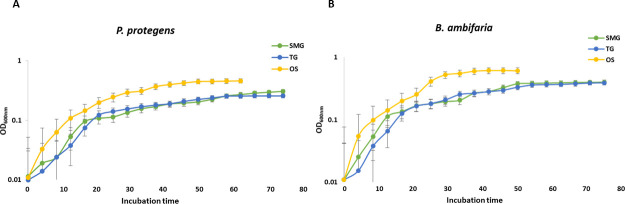
Simulated microgravity affects the growth rate of *P. protegens* and *B. ambifaria*. (**A**) Growth curve of (**A**) *P. protegens* and (**B**) *B. ambifaria* under simulated microgravity (SMG), traditional gravity (TG), and orbital shaking (OS) control. X-axis values represent hours.

Experiments here used a well-established Rotary Cell Culture System (RCCS) equipped with High-Aspect Rotating Vessels (HARVs), also known as clinostats (Synthecon), that simulate the effects of microgravity on Earth (35, 36, 39). Clinostats create a simulated weightlessness state by rotating around one axis to negate the effects of gravity (clino-rotation principle) (35, 39). In this system, the horizontal rotational axis (perpendicular to the gravity vector) simulates microgravity (SMG), while the vertical rotational axis (parallel to the gravity vector) permits traditional gravity (TG) (36, 38, 40, 41) (Fig. 1). To assess the impact of SMG and TG on the growth rate of both *P. protegens* and *B. ambifaria* within the clinostat vessels, OD₆₀₀ of cultures was measured every 4 h for 72 h (Fig. 2A). For *P. protegens*, a brief lag phase of ≤5 h was observed under both SMG and TG conditions in the clinostat. The growth rate during the exponential phase remained comparable between SMG and TG, with cultures beginning to show slowed growth at approximately 25–30 h of incubation, representing the late exponential phase. In contrast, *B. ambifaria* exhibited an earlier onset of the exponential phase, achieved higher overall biomass, and began slowing growth earlier at approximately 20–25 h (Fig. 2B). The fact that both organisms have similar growth parameters between SMG and TG cultivation means that meaningful physiological differences observed between the two conditions can be attributed largely to the altered perceived gravity. Although both strains showed similar growth patterns under SMG and TG conditions, both organisms seemed to display slower growth rates and lower overall biomass in clinostat cultures compared to cultures cultivated in tubes in an orbital shaker (OS). This difference could be influenced by factors such as reduced mixing or oxygen availability within the clinostat vessels. Based on these growth profiles, two time points were selected for downstream analyses. The Day 1 (24 h) time point was chosen to represent the exponential phase; although this time point is in the late exponential phase, it was necessary to use a later time point so that sufficient biomass was produced to capture metabolites. Day 3 (72 h) was chosen to represent the stationary phase.

### Simulated microgravity affects the production of plant-promoting secondary metabolites

**Fig 3:**
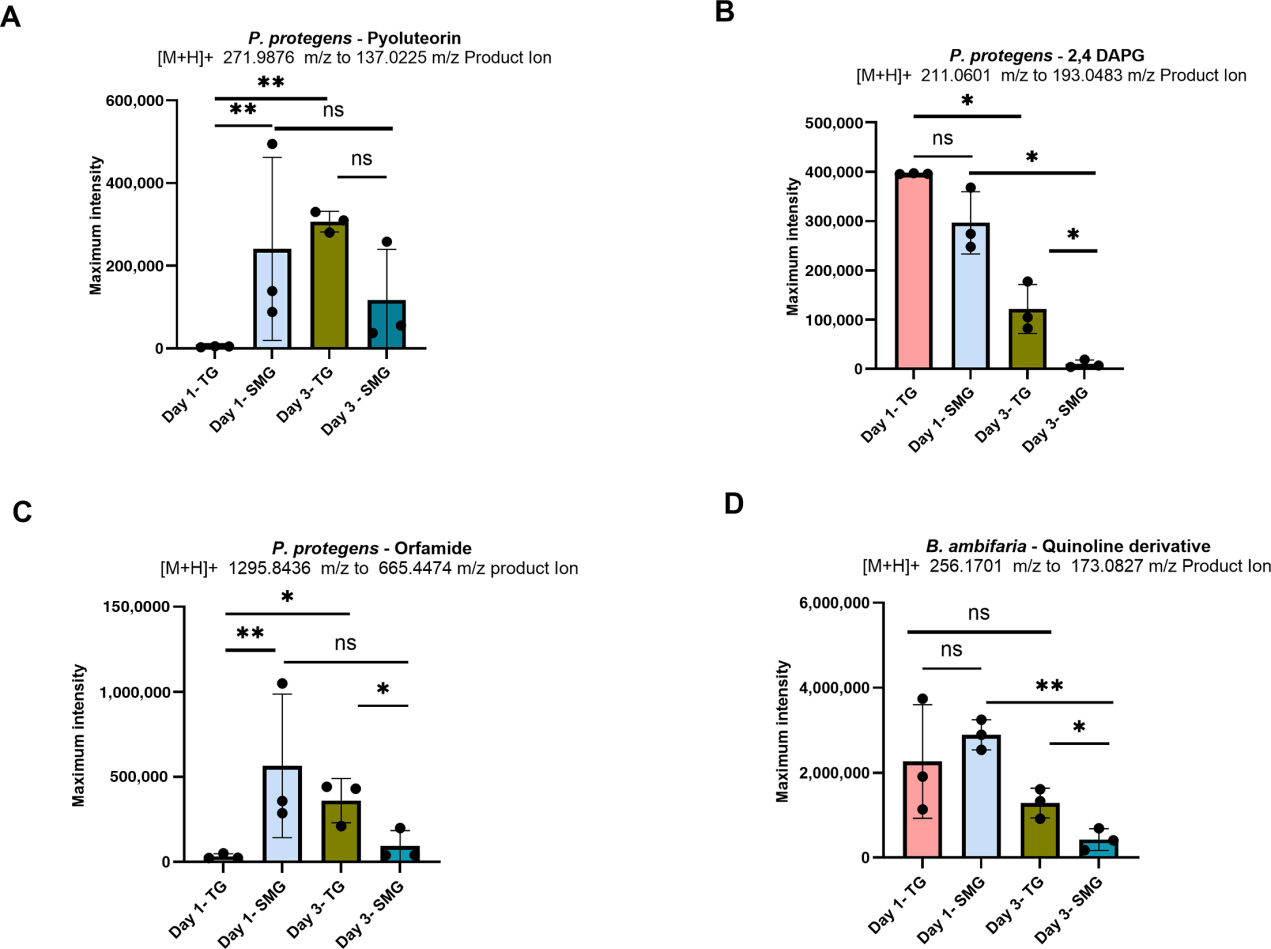
Relative production of select plant-promoting secondary metabolites under traditional gravity and simulated microgravity in *P. protegens* and *B. ambifaria*. (**A**) Pyoluteorin levels, (**B**) 2,4-DAPG levels, and (**C**) orfamide A levels on Day 1 and Day 3 in *P. protegens* cultured in TG and SMG conditions. (**D**) PQQ levels in *B. ambifaria* cultured in TG and SMG conditions on Day 1 and Day 3. Metabolite levels were measured via LC-MS/MS and are shown as maximum intensity values (*n* = 3 biological replicates per condition). Bars show mean ± SD of three biological replicates (*n* = 3), normalized to OD₆₀₀. Statistical significance was determined using a two-tailed Welch’s *t*-test; *P* < 0.05 (*), *P* < 0.01 (**), ns = not significant.

Relative quantification of pyoluteorin, 2,4-diacetylphloroglucinol (2,4-DAPG), and orfamide A in *P. protegens* and pyrroloquinoline in *B. ambifaria* was performed using targeted LC-MS/MS at both 24-h (Day 1) and 72-h (Day 3) time points under SMG and TG conditions. Day 1 and Day 3 time points were chosen as representative time points of late exponential and stationary phase cultures, respectively. Targeted LC-MS/MS acquisition was run on the predicted specific *m/z* values for each compound, from which a fragment mass could be used for relative quantification. In *P. protegens*, pyoluteorin at *m/z* 271.9876 (retention time [RT]: 6.83 min, ΔRT 1.0, collision energy [CE] 25, fragment at 137.0225 *m/z*), 2,4-DAPG was detected at *m/z* 211.0601 (RT: 7.72 min, ΔRT 1.0, CE 25, fragment at 193.0483 *m/z*), and orfamide A at *m/z* 1295.8436 (RT: 11.63 min, ΔRT 1.0, CE 53.89, fragment at 665.4474 *m/z*) (Fig. S1A). In *B. ambifaria*, pyrroloquinoline was detected at *m/z* 256.1701 (RT 8.24 min, ΔRT 0.75, CE 35.29, 173.0827 *m/z*) (Fig. S2). Relative quantification of targeted secondary metabolites was performed between SMG and TG conditions. This experiment was based on the mean of replicates and normalized to OD₆₀₀ to account for biomass differences. In *P. protegens*, pyoluteorin exhibited significantly higher yield (*P* < 0.05) under SMG at Day 1 compared to TG, but yield decreased by Day 3, and this change was not statistically significant (*P* = 0.0763). In contrast, TG cultures showed a significant increase in pyoluteorin accumulation from Day 1 to Day 3 (*P* < 0.05), while the decrease in SMG over time remained not statistically significant (*P* = 0.4580), indicating divergent temporal regulation of this metabolite (Fig. 3A; Table S1); 2,4-DAPG showed consistently lower yields under SMG compared to TG at both time points, with the difference at Day 1 not reaching statistical significance (*P* = 0.11), whereas the decrease observed at Day 3 was statistically significant (*P* < 0.05) (Fig. 3B). Both TG and SMG cultures exhibited a significant decrease in production over time (*P* < 0.05) (Fig. 3B). A trend similar to pyoluteorin was observed for orfamide A, which showed a statistically significant (*P* < 0.05) increase under SMG at Day 1, followed by a statistically significant decrease by Day 3 (*P* < 0.05), while TG conditions supported sustained or increased levels (Fig. 3C). Under TG, orfamide production increased significantly from Day 1 to Day 3 (*P* < 0.05). In contrast, SMG cultures remained comparable over time (*P* = 0.1874) (Fig. 3C). In *B. ambifaria*, relative quantification of pyrroloquinoline revealed higher production under SMG at Day 1, although the difference compared to TG was not statistically significant (*P* = 0.4), followed by a statistically significant decline by Day 3 (Fig. 3D; Table S1). Production decreased significantly over time (*P* < 0.05), with a more pronounced reduction under SMG, compared to a non-significant change under TG (*P* = 0.307) (Fig. 3D). Thus, while most compounds show elevated early production in SMG, 2,4-DAPG and the quinolone derivative exhibited decreased production at later time points, whereas pyoluteorin and orfamide A remained comparable between Day 1 and Day 3 under SMG. In the context of prolonged plant cultivation, such reductions in metabolite output during the stationary phase could pose challenges to sustained biocontrol activity. Therefore, assessing the effects of extended microgravity exposure is essential to distinguish between short-term stress responses and true adaptive shifts in bacterial physiology. Prior studies suggest that long-term exposure to low-shear simulated microgravity induces physiological adaptations not evident during early growth stages (42). Accordingly, the 72-h (Day 3) time point used throughout most of this study is intended to capture key aspects of longer-term adaptation, providing a more representative understanding of bacterial behavior under SMG conditions.

### Simulated microgravity showed altered expression of genes associated with metabolism in plant-beneficial bacteria

To investigate differential gene expression under SMG, global transcriptomic profiles of *P. protegens* and *B. ambifaria* were compared between SMG and TG conditions using RNA-seq. In *P. protegens* at Day 1, a total of 1,412 genes were differentially expressed under SMG compared to TG when applying a statistical cutoff of *P* < 0.05. Of these, 670 genes were upregulated, and 791 were downregulated. Applying a more stringent dual cutoff (*P* < 0.05 and >1.5-fold change), 345 genes were upregulated, and 401 genes were downregulated under SMG relative to TG (Table S2). At Day 3, 2,958 genes were differentially expressed under SMG at *P* < 0.05, with 1,495 genes upregulated and 1,463 downregulated. When applying the dual cutoff of *P* < 0.05 and >1.5-fold change, 955 genes were upregulated, and 250 genes were downregulated in SMG compared to TG (Table S2).

For *B. ambifaria*, 2,190 genes were differentially expressed under SMG compared to TG at Day 1, using a single cutoff of *P* < 0.05. Among these, 1,040 genes were upregulated, and 1,149 were downregulated. Applying the dual cutoff (*P* < 0.05 and >1.5-fold change), 594 genes were upregulated, and 910 genes were downregulated on Day 1 (Table S3). At Day 3, 1,259 genes were differentially expressed under SMG (*P* < 0.05), including 725 upregulated and 534 downregulated genes. Under the dual cutoff, 620 genes were upregulated, and 629 genes were downregulated compared to TG (Table S3).

**Fig 4:**
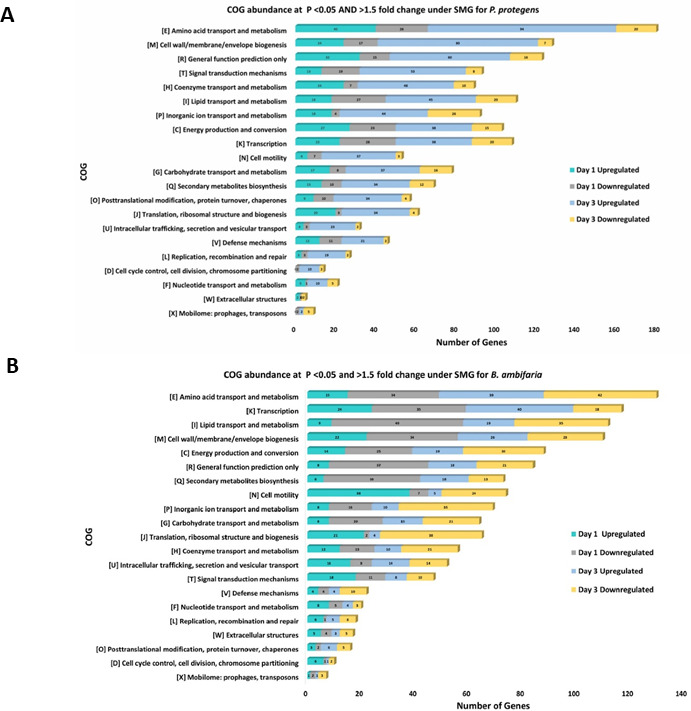
COG abundance of significantly differentially expressed genes at two cutoffs: *P* < 0.05 and >1.5-fold change under SMG conditions for *P. protegens* and *B. ambifaria*. Bar plots display the number of genes assigned to each COG category that were significantly differentially expressed under SMG compared to TG (fold change > 1.5, *P* < 0.05) for (**A**) *P. protegens* and (**B**) *B. ambifaria*. Data represent three biological replicates (*n* = 3). The y-axis indicates the COG categories, and the x-axis represents the number of differentially expressed genes. Color coding reflects both the timing of regulation and regulation pattern: cyan = Day 1 upregulated, gray = Day 1 downregulated, light blue = Day 3 upregulated, and yellow = Day 3 downregulated.

Genes were categorized into Clusters of Orthologous Groups (COG) to identify overrepresented functional categories among the differentially expressed genes. In both *P. protegens* and *B. ambifaria*, the majority of altered genes at both Day 1 and Day 3 were associated with metabolism**,** transcription**,** cell wall biogenesis, and energy production. These categories exhibited dynamic regulatory changes over time, suggesting broad physiological adaptation in response to SMG conditions (Fig. 4). Briefly, in *P. protegens,* genes involved in amino acid metabolism showed marked upregulation over time, with the highest number of significantly upregulated genes (*P* < 0.05) observed by Day 3. This pattern may reflect oxidative stress responses, as amino acids like proline support redox balance and protect against abiotic stress (20) or potentially increased biosynthetic demand or metabolic adaptation. Similarly, genes associated with lipid metabolism, cell wall/membrane biogenesis**,** and inorganic ion transport and metabolism were substantially upregulated at Day 3, perhaps suggesting efforts to maintain membrane fluidity in response to altered shear under SMG. Notably, carbohydrate transport and metabolism genes also showed an increase in upregulation from Day 1 to Day 3. Additionally, the high abundance of genes related to transcriptional activity, which intensified over time, further indicates a global regulatory shift to accommodate long-term exposure to SMG conditions. Furthermore, genes involved in secondary metabolite biosynthesis exhibited notable transcriptional changes across both time points, with marked upregulation at Day 1 and downregulation observed at Day 3. Genes related to signal transduction and energy production also showed significant upregulation, suggesting active environmental sensing and a likely increase in energy demand to support enhanced cellular maintenance under SMG conditions (Fig. 4A).

In *B. ambifaria,* transcriptomics profiling revealed similar global transcriptional changes with notable shifts in gene regulation between Day 1 and Day 3. Categories such as amino acid and lipid metabolism undergo early upregulation but are then followed by subsequent downregulation, standing in stark contrast to the *P. protegens* results and highlighting the individuality of bacterial SMG responses. Transcription-related genes were initially downregulated at Day 1 but became upregulated by Day 3, indicating a delayed regulatory adjustment. Genes involved in cell wall/membrane biogenesis and energy production also showed increased upregulation at Day 3, suggesting elevated metabolic demand as part of long-term adaptation to SMG conditions, as mentioned before. Genes related to secondary metabolite biosynthesis showed a shift in expression patterns over time, with a mixed response at both time points. At Day 1, the majority of genes were downregulated, with a smaller subset showing upregulation, whereas by Day 3, both up- and down-regulated genes were observed, reflecting fluctuation in secondary metabolite production under prolonged SMG exposure. Interestingly, in *B. ambifaria*, cell motility genes were also upregulated at Day 1, likely as an early stress response, but sharply downregulated by Day 3, suggesting a suppression of motility with longer-term adaptation to SMG (Fig. 4B).

Together, these patterns illustrate a coordinated, time-dependent transcriptional response to simulated microgravity. Early-phase transcriptional adjustments transition into sustained activation of key metabolic, structural, and regulatory pathways, reflecting a broader adaptation strategy to this environment. Given that primary and secondary metabolic pathways are interlinked, where products from primary metabolism are channeled into secondary metabolite biosynthesis (20), the observed altered regulation of genes involved in amino acid**,** lipid, and carbohydrate metabolism under microgravity likely impacts secondary metabolism. This connection is supported by global transcriptomic data showing broad changes in primary metabolic gene expression. To further investigate the compounds with fluctuating yields identified through metabolomics, the corresponding biosynthetic gene clusters were specifically examined, and their transcriptional activity was correlated with the observed changes in metabolite yields.

### Integrated transcriptomic/metabolomic analysis links downregulated gene expression to reduced orfamide A and pyrroquinolone production

**Fig 5:**
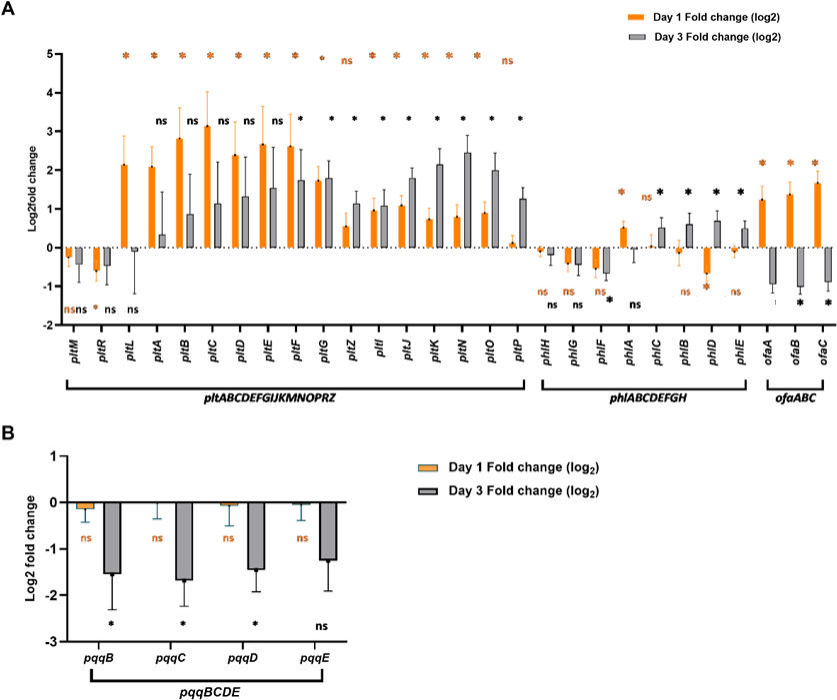
Transcriptional changes in biosynthetic clusters under SMG via RNA-seq. (**A**) Log₂ fold change in gene expression of the *plt*, *phl*, and *ofa* biosynthetic clusters in *P. protegens* at Day 1 (orange bars) and Day 3 (gray bars) under SMG relative to TG. Significant upregulation was observed for *ofaABC* and *plt* genes at Day 1, while the expression declined or became variable by Day 3. Genes of the *phl* cluster showed mixed regulation, with some genes significantly downregulated at Day 3. (**B**) Log₂ fold change in expression of the *pqqBCDE* cluster in *B. ambifaria*. Expression changes were not significant at Day 1 but showed strong downregulation for most genes by Day 3 under SMG. Bars represent mean ± SD (*n* = 3) of biological replicates. Statistical significance was determined using a two-tailed Welch’s *t*-test; *P* < 0.05 (*), *P* < 0.01 (**), ns = not significant.

To specifically address the impact of SMG on plant-promoting secondary metabolites, the RNA-seq data were analyzed for the expression of biosynthetic gene clusters responsible for the production of pyoluteorin, 2,4-diacetylphloroglucinol (DAPG), and orfamide in *P. protegens*, and pyrroquinoline in *B. ambifaria*. Differential expression was compared at both 24-h (Day 1) and 72-h (Day 3) incubation time points to capture the exponential and stationary phase effects. At the Day 1 time point, 15 out of the 17 genes in the pyoluteorin biosynthetic gene cluster (*pltABCDEFGIJKMNOPRZ*) in *P. protegens* were upregulated under SMG, each exhibiting >1.5-fold change. These genes included *pltA, pltB, pltC, pltD, pltE, pltF, pltG, pltZ, pltI, pltJ, pltK, pltN, pltO, pltP*, and *pltL*. Of these, 13 genes met the statistical significance threshold (*P* < 0.05), with *pltP* and *pltZ* being the exceptions (Fig. 5A; Table S2). Two genes within the cluster, *pltM* and *pltR*, were downregulated under SMG. While *pltM* did not meet the fold change or statistical significance cutoffs, *pltR* showed significant downregulation with a >1.5-fold change and *P* < 0.05. The overall increase in transcription is consistent with increased pyoluteorin detected in SMG samples (Fig. 3A). However, at the 72-h (Day 3) time point, expression data and pyoluteorin production did not correlate. An expression pattern similar to Day 1 was observed in the pyoluteorin biosynthetic gene cluster; 14 out of the 17 genes were upregulated under SMG, including *pltA****,*** *pltB****,*** *pltC****,*** *pltD****,*** *pltE****,*** *pltF****,*** *pltG****,*** *pltZ****,*** *pltI****,*** *pltJ****,*** *pltK****,*** *pltN****,*** *pltO*, and *pltP* (Fig. 5A; Table S2). All genes except *pltA* showed a >1.5-fold change, and nine genes (*pltF****,*** *pltG****,*** *pltZ****,*** *pltI****,*** *pltJ****,*** *pltK****,*** *pltN****,*** *pltO*, and *pltP*) met the statistical significance threshold of *P* < 0.05. In contrast, three genes—*pltM*, *pltR*, and *pltL*—were downregulated, but none met the fold-change or significance criteria (Fig. 5A). While the overall gene expression of this cluster was increased in SMG compared to TG, there was no statistical difference in pyoluteorin production (Fig. 3A).

In the 2,4-DAPG biosynthetic gene cluster (*phlABCDEFGH*) of *P*. protegens, on Day 1, six out of eight genes (*phlB****,*** *phlD****,*** *phlE****,*** *phlH****,*** *phlG****,*** and *phlF*) were downregulated under SMG. Among these, only *phlD* satisfied both the >1.5-fold change and *P* < 0.05 significance thresholds (Fig. 5A; Table S2). In contrast, the remaining two genes, *phlA* and *phlC*, were upregulated. However, only *phlA* showed a statistically significant difference (*P* < 0.05), despite having a fold change below 1.5. The overall lack of change in expression is consistent with no statistically significant difference in 2,4-DAPG production on Day 1 (Fig. 3B). On Day 3, four genes, *phlH****,*** *phlG****,*** *phlF*, and *phlA*, were downregulated, but only *phlF* showed a >1.5-fold change and met the statistical significance threshold (*P* < 0.05). The remaining four genes, *phlC, phlB, phlD,* and *phlE,* were upregulated. Among them, *phlB* and *phlD* showed >1.5-fold change, and all four met the *P* < 0.05 threshold. Despite these, 2,4-DAPG production was nearly undetectable on Day 3, which was a statistically significant decrease from TG on Day 3 (Fig. 3B). Here, again, the transcriptional changes do not strongly correlate with the metabolite production.

In contrast, better correlation was seen for the orfamide A biosynthetic cluster (*ofaABC*). All three genes involved (*ofaA****,*** *ofaB***,** and *ofaC*), which encode the arthrofactin-type cyclic lipopeptide synthetase subunits A, B, and C, respectively (43), were significantly upregulated under SMG on Day 1 (Fig. 5A; Table S5). Each gene exhibited a >1.5-fold change with *P* < 0.05, indicating coordinated transcriptional activation of the entire orfamide biosynthetic cluster. This correlated to a statistically significant increase in orfamide A detection in SMG on Day 1 compared to TG (Fig. 3C). Interestingly, all three genes in the orfamide biosynthetic gene cluster were significantly downregulated on Day 3. Each gene exhibited a >1.5-fold decrease in expression (greater than 50% reduction relative to TG), with *P* < 0.05 for all three genes (Fig. 5A; Table S2). This downregulation correlates to the statistically significant decrease in orfamide A detection on Day 3 in SMG (Fig. 3C). Therefore, for orfamide A, gene expression patterns match metabolite production patterns, suggesting that gene transcription may be a major method of control for the production of this compound.

Similar results were obtained for PQQ biosynthesis in *B. ambifaria*. At Day 1, all four genes in the *pqqBCDE* biosynthetic cluster*, pqqB, pqqC, pqqD*, and *pqqE*, were slightly downregulated in SMG, but none met the thresholds for fold change or statistical significance (Fig. 5B; Table S3). This correlates to no statistical difference in PQQ detection at that time (Fig. 3D). On Day 3, all four genes were again found to be downregulated under SMG, but this time, *pqqB, pqqC*, and *pqqD* exhibited >1.5-fold change and met the statistical significance threshold (*P* < 0.05), while *pqqE*, which encodes the PqqA peptide cyclase, showed >1.5-fold change but did not show statistical significance (*P* = 0.55). This stronger Day 3 downregulation correlates with a statistically significant decrease in PQQ detection in SMG (Fig. 3D). These findings suggest time-dependent transcriptional repression of the *pqq* cluster under SMG, potentially impacting quinolone biosynthesis. Similarly, the consistent downregulation of all four genes in the *pqqBCDE* operon under simulated microgravity indicates strong operon-level regulation. A summary of these observations is provided in Table 1.

**TABLE 1:** Summary of correlated transcriptomics and metabolomics levels for Day 1 and Day 3 in SMG

Metabolites	Day 1 (RNA-seq)	Day 1 (MS)	Day 3 (RNA-seq)	Day 3 (MS)
Pyoluteorin	15 up- and 2 down-regulated	High yield	14 up- and 2 down-regulated	Low yield
2,4-DAPG	6 down- and 2 up-regulated	Low yield	4 up- and 4 down-regulated	Low yield
Orfamide A	Upregulated	High yield	Downregulated	Low yield
PQQ	No change	High yield	Downregulated	Low yield

**Fig 6:**
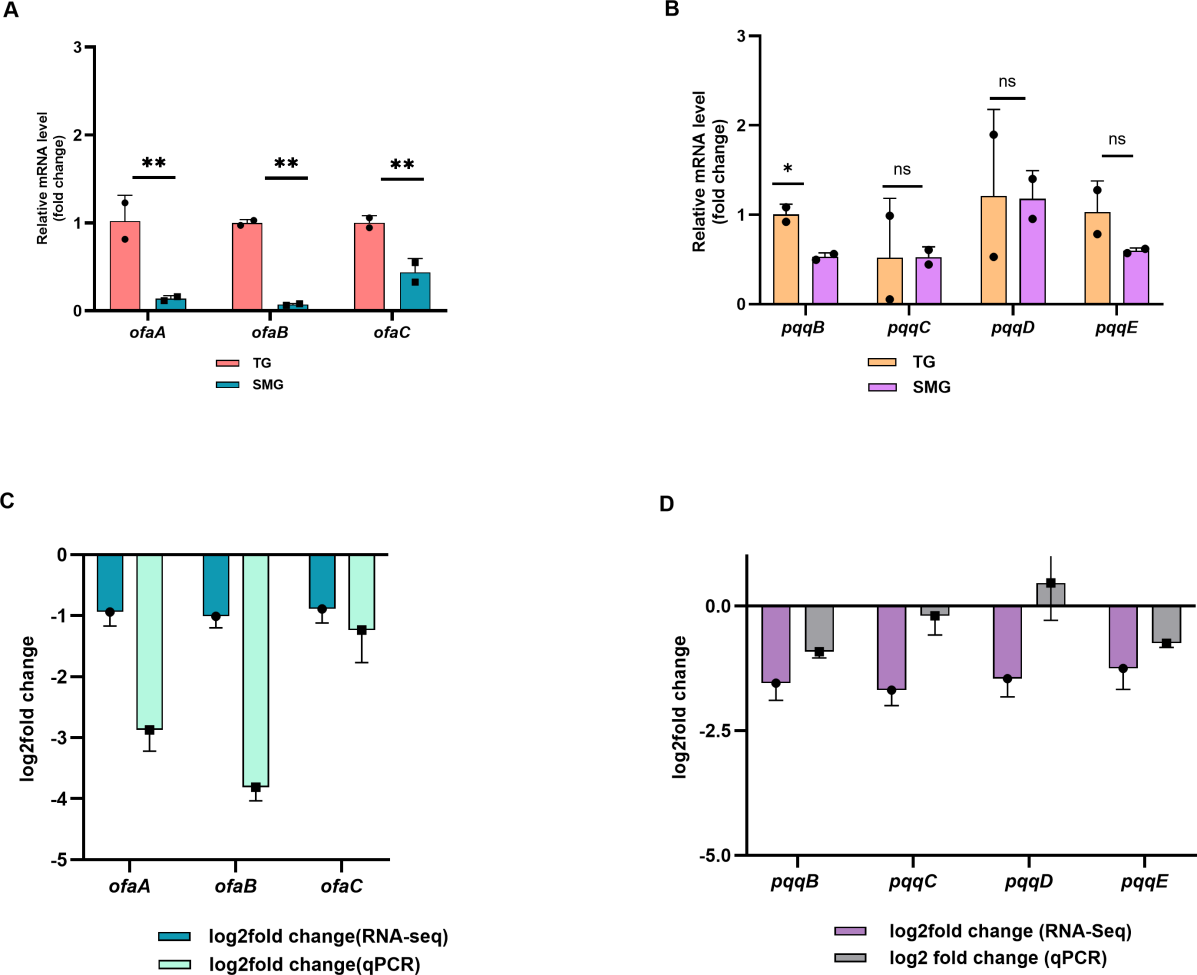
Validation of transcriptomic results for orfamide and quinoline biosynthetic genes using RT-qPCR. (**A**) Relative mRNA levels of *ofaA*, *ofaB*, and *ofaC* in *P. protegens*. All three *genes* showed significant downregulation under SMG. (**B**) Relative mRNA expression levels of *pqqB*, *pqqC*, *pqqD*, and *pqqE* in *B. ambifaria*. Significant downregulation was observed for *pqqB,* while expression changes in *pqq*C, *pqqD,* and *pqqE* were not significant. Bars show mean ± SD of two biological replicates (*n* = 2) with two technical replicates. Statistical significance was determined using a two-tailed Welch’s *t*-test; *P* < 0.05 (*), *P* < 0.01 (**), ns = not significant. (**C, D**) log₂ fold change values for gene expression from RNA-seq (mean of three biological replicates) and RT-qPCR (mean of two biological replicates, each with two technical replicates) for *ofaABC* (**C**) and *pqqBCDE* (**D**), showing consistent downregulation across both methods. Data represent mean ± SD from the respective replicates. These panels illustrate the similarity in expression trends between RNA-seq and qPCR.

The decreased production of plant-promoting secondary metabolites in the stationary phase is problematic for the use of these organisms in space-faring plant cultivation. With the aim of genetically engineering strains to increase the production of important compounds, further analysis was targeted at compounds that show the strongest correlation between transcriptional activity and yield, as these compounds are the ones most likely to be affected by simple genetic manipulation, that is, they may not require more complicated metabolic engineering. These compounds of interest were orfaminde A in *P. protegens* and PQQ in *B. ambifaria*. As a first step to genetic engineering, RT-qPCR was performed to validate the directionality and relative magnitude of gene expression changes observed in the Day 3 RNA-seq data set. In *P. protegens*, *rpsA* was used as a reference gene for normalization, as its expression was consistent across all SMG replicates. RT-qPCR analysis revealed that all three genes in the *ofaABC* operon were downregulated under SMG on Day 3 (Fig. 6A). Each gene exhibited >1.5-fold downregulation in SMG (Table S4). In fact, the RT-qPCR results suggested even more dramatic downregulation of these genes (Fig. 6C). Together, these results support the conclusion that SMG induces a robust transcriptional suppression of the *ofaABC* operon rather than contradicting the RNA-seq findings. Given that *ofaA* is critical for orfamide synthesis and *ofaB/C* contribute to overall yield (44), the transcriptional suppression observed under SMG correlates to reduced orfamide production detected at the metabolite level.

Similarly, RT-qPCR analysis of *pqqB, pqqC, pqqD*, and *pqqE* showed that all four genes showed reduced expression under SMG (Fig. 6B; Table S4), although not all differences reached statistical significance. Among these, the first gene in the operon *pqqB* exhibited the greatest downregulation, with >1.5-fold change and statistical significance (*P* = 0.0208). The *pqqC* and *pqqE* genes also showed reduced expression (1.14-fold and >1.5-fold, respectively), while *pqqD* showed a modest 1.13-fold upregulation, although the differences were not statistically significant (*pqqC* [*P* = 0.9939], *pqqD* [*P* = 0.9673], and *pqqE* [*P* = 0.2215]). While these results were less dramatic than the RNA-seq results (Fig. 6D), the consistent directionality across platforms strengthens confidence in the observed transcriptional response under SMG. Importantly, these RT-qPCR results corroborated RNA-seq-derived expression trends and provide targeted support for genes of functional interest. The overall RT-qPCR trends were consistent, suggesting that genetic manipulation may impact biosynthesis and yield of these metabolites.

### Identification of transcriptional start sites of highly expressed genes and characterization of P*_glnA_2_* as a strong SMG promoter in *P. protegens*

One method for correcting the lack of orfamide A production under SMG is to replace its promoter with one that shows strong expression in SMG. To implement such a strategy, the RNA-seq data were analyzed to identify genes highly expressed in *P. protegens* under SMG. In RNA-seq analysis, a gene’s transcriptional activity is positively correlated with its reads per kilobase per million mapped reads (RPKM) (45, 46). RPKM normalizes read counts by gene length and total mapped reads to reflect transcript abundance. All *P. protegens* genes were ranked by raw read counts (47), and corresponding RPKM values were calculated to compare expression levels. The top 30 highly expressed genes were ranked by RPKM values (Table S5). Ribosomal protein-encoding genes, which are typically highly expressed due to their essential roles in translation and cell growth (48), were largely excluded to filter out general expression patterns that are not condition-specific. However, *rpsA* was retained as a representative ribosomal gene to assess whether its expression was relatively higher under microgravity conditions, given its consistent expression across at least two biological replicates.

**Fig 7:**
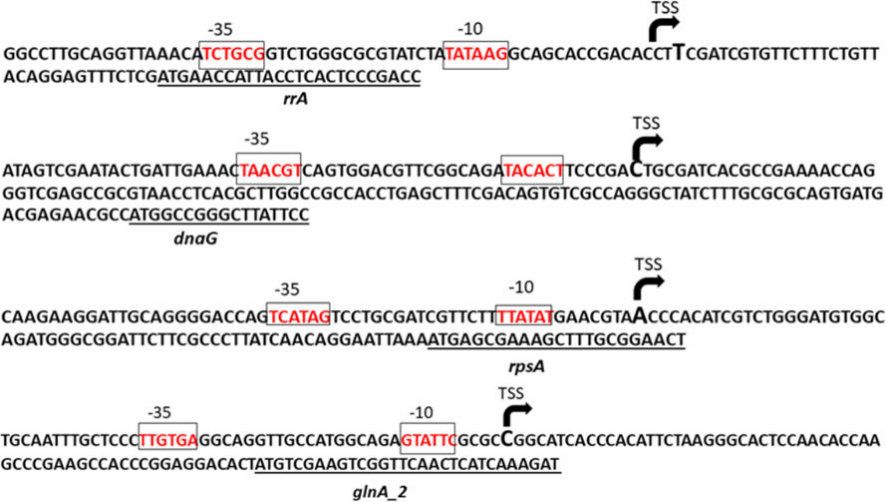
Identification of putative promoter sequences and transcription start sites (TSS) upstream of *rrA, rpoD*, *rpsA,* and *glnA_2* genes in *P. protegens*. Transcriptional start sites upstream of each gene were identified by 5′ RACE, indicated by arrows. Predicted –35 and –10 elements of the σ⁷⁰ promoter are boxed and labeled. Optimal 16–18 nt spacing for canonical promoters was observed in each, except for suboptimal 19 nt spacing for *glnA_2* spacer between the −35 and −10 elements.

Ultimately, four candidates (*oprF***,** *rpsA***,** *glnA_2*, and *rpoD*) were selected based on reproducible expression patterns supported by both RPKM and raw read count data. To locate their respective promoter regions, transcriptional start sites (TSS, +1) were identified using 5′ RACE. Sequencing of the 5′ RACE products revealed the TSS for each gene: 60 bp upstream of the start codon for *glnA₂*, 61 bp for *rpsA*, 113 bp for *rpoD,* which is located in the same operon as *dnaG* and transcribed from the *dnaG*-initiated transcript, and 33 bp for *oprF*, which is part of the *rrA*-initiated operon (Fig. 7). These TSS positions were used as reference points to define the corresponding promoter regions. A motif resembling the σ^70^-dependent extended −10 element was present upstream from each identified TSS. A −35-like element resembling the σ^70^ consensus sequence was predicted 17–18 bp upstream of the −10 element for *rpsA*, *rpoD,* and *oprF*, which is considered optimal (49–52). In contrast, for the *glnA_2* promoter, a slightly longer 19 bp spacer was identified, which is considered suboptimal (53). The predicted promoter elements were further validated using multiple promoter prediction tools, including BDGP (45), iPromoter-2L (54), and Sapphire (55). These prediction tools generated similar promoter elements as the 5’ RACE analysis (Fig. S2).

**Fig 8:**
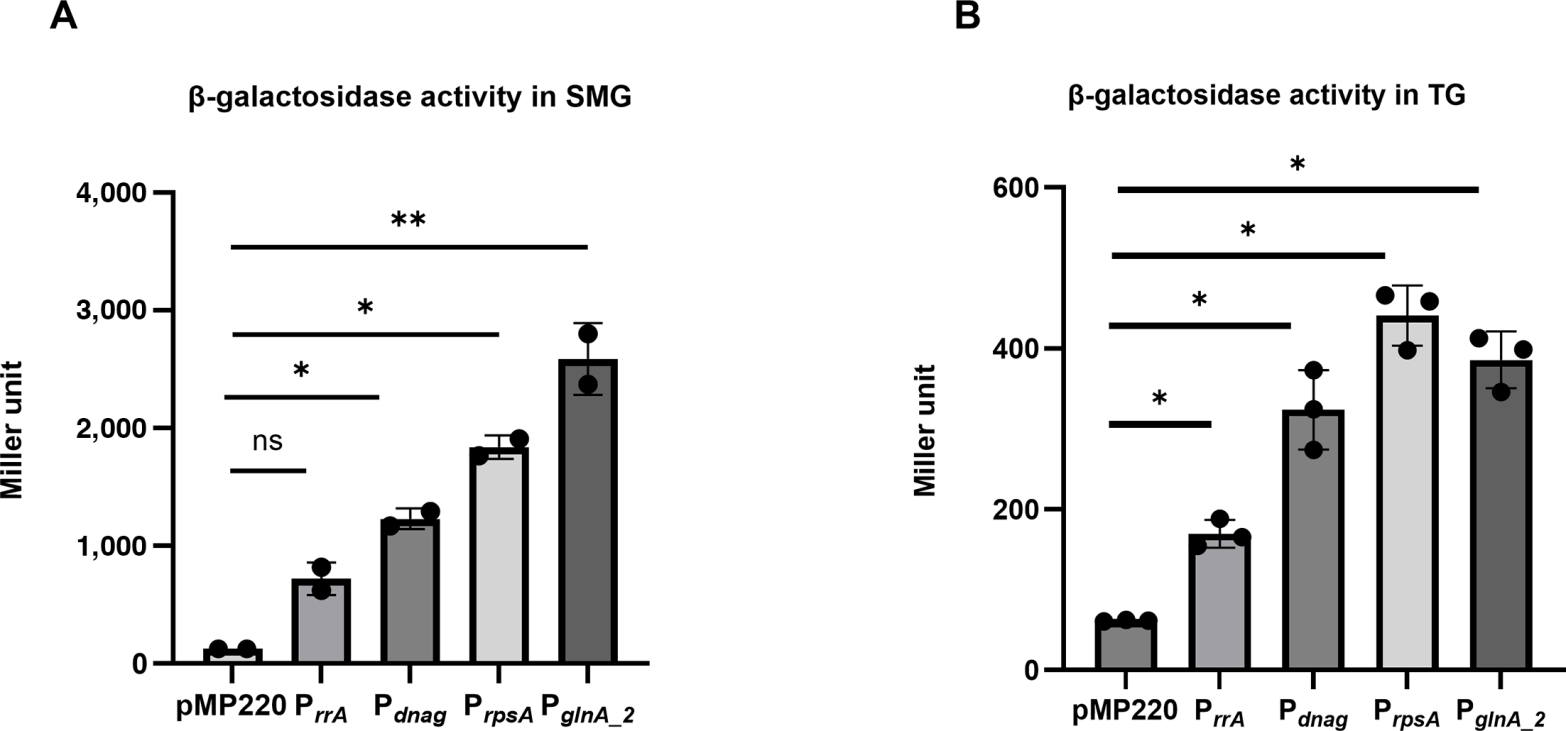
β-galactosidase activity reporter constructs in *P. protegens* under SMG and TG. Bar graph depicts Miller units of β-galactosidase activity for *lacZ* reporter constructs under the control of different promoters: pMP220 (vector control), P*_rrA_-lacZ*, P*_dnaG_-lacZ*, P*_rpsA_-lacZ*, and P*_glnA_2_-lacZ* under (**A**) SMG and (**B**) TG. Bars represent mean ± SD (*n* = 6). Statistical significance was determined using a two-tailed Welch’s *t*-test; *P* < 0.05 (*), *P* < 0.01 (**), ns = not significant.

Promoter activity was assessed by constructing transcriptional fusions of each promoter region to a promoterless *lacZ* gene in the pMP220 plasmid. β-galactosidase assays were conducted in *P. protegens* with each construct under SMG, and promoter activities were measured after 72 h of incubation. Among the four tested promoters, P*_glnA_2_* exhibited the highest activity at 2,587 ± 371 MU (1), followed by P*_rpsA_*, which yielded around 1,837 ± 299 MU (Fig. 8). Promoters P*_rpoD_* and P*_rrA_* showed moderate activity, with values of 1,229 ± 196 MU and 720 ± 121 MU, respectively (Fig. 8A). In contrast, the negative control strain with empty pMP220 displayed minimal basal activity at roughly 127 ± 5 MU. These results demonstrated that P_g_*_lnA₂_* and P*_rpsA_* exhibit the strongest activity under simulated microgravity, with statistically significant differences in activity levels, and are therefore promising candidates for driving robust gene expression when replacing the native P*_ofaABC_* promoter. To determine whether the promoter activity was specific to SMG or indicative of strong constitutive expression, β-galactosidase assays were performed under TG using each promoter construct. Under TG conditions, promoter activity was observed in the following order: P*_rpsA_* (440 ± 42 MU) > P*_glnA_* (385 ± 30 MU) > P*_rpoD_* (323 ± 36 MU) > P*_rrA_* (169 ± 22 MU) (Fig. 8B). This trend closely mirrors the pattern observed under SMG, with the exception that P*_rpsA_* exhibited slightly stronger activity than P*_glnA_2_* under TG, which was reversed in the SMG condition. These results support the conclusion that all four promoters possess strong constitutive activity, although their relative strength may be modulated by SMG. Furthermore, the orders of promoter strength reflected by the reporter assays were observed to be identical to the results obtained from RNA-seq (read count) in both SMG and TG, which demonstrated that the results of transcriptome sequencing analysis and promoter activity experiments were highly consistent.

### Promoter replacement leads to significant transcriptional upregulation under SMG

In an effort to induce orfamide A synthesis under SMG, the native *ofaABC* promoter (44) was replaced with the P*_glnA_2_* promoter. As an alternative, the native *ofaABC* promoter was also replaced with the well-characterized inducible promoter P_BAD_ (56). P_BAD_ is controlled by the AraC regulator, which activates transcription in the presence of L-arabinose (56, 57). Importantly, *P. protegens* can take up L-arabinose but is unable to catabolize it (58, 59), ensuring sustained inducer availability without rapid consumption. This feature makes P_BAD_ especially suitable for precise and tunable gene expression (57). To induce PQQ synthesis in *B. ambifaria*, a third strategy was employed: the entire *pqqBCDE* operon was placed under the control of an *l*-rhamnose-inducible P_Rha_ promoter on a replicating plasmid. This promoter is commonly used in *Burkholderia* species due to its tight regulation. Although the specific mechanism for rhamnose uptake and utilization in AMMD is not confirmed, the system has been effective in related species (60). This strategy was chosen to allow inducible and tunable overexpression while avoiding the complexity of chromosomal integration in a less genetically tractable *Burkholderia* species (61). Additionally, the *pqqBCDE* operon is under complex regulation, influenced by quorum-sensing systems and environmental conditions (62). Placing the operon on a plasmid with an inducible promoter removes it from any complicating chromosomal context.

**Fig 9:**
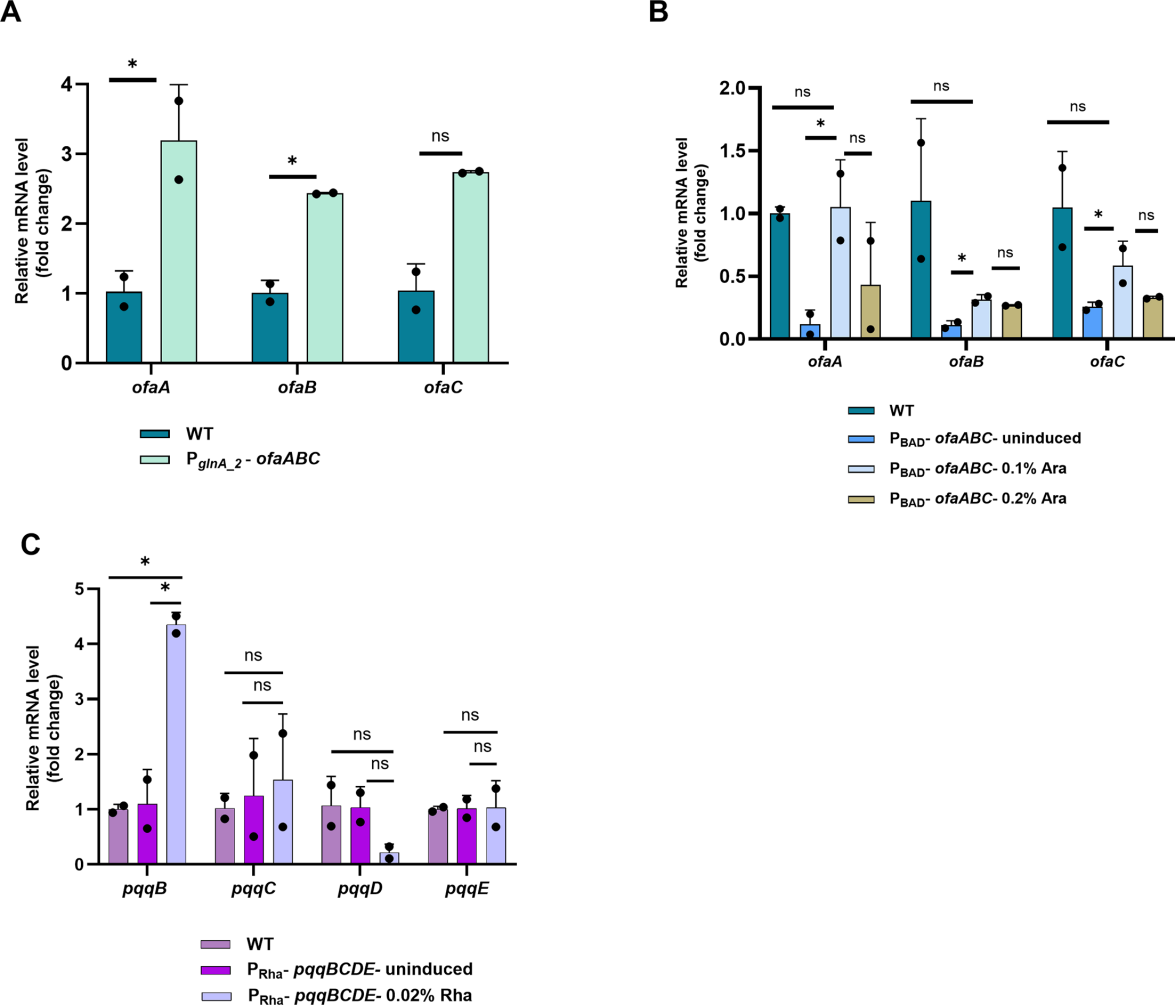
Transcriptional analysis of replaced promoter strains of *ofaABC* in *P. protegens* and the *pqqBCDE* operon in *B. ambifaria*. (**A**) Comparative expression of *ofaA*, *ofaB*, and *ofaC* between WT and P*_glnA_2_-ofaABC*. Significant differences were observed in the transcript levels of *ofaA*. (**B**) Effect of 0.1% and 0.2% arabinose induction on P_BAD_-*ofaABC* expression in *P. protegens*. Induction with 0.1% arabinose significantly increased *ofaA*, *ofaB,* and *ofaC* transcript levels (*P* < 0.05). At 0.2% induction, no genes were significantly induced. (**C**) Relative mRNA expression of *pqqB*, *pqqC*, *pqqD*, and *pqqE* in *B. ambifaria* P*_rha_- pqqBCDE* under uninduced and 0.02% rhamnose-induced conditions. Induction significantly upregulated *pqqB* expression, while other genes in the operon remained unchanged. Bars represent mean ± SD from two biological and two technical replicates for each biological replicate. Statistical significance was determined using a two-tailed Welch’s *t*-test; *P* < 0.05 (*), *P* < 0.01 (**), ns = not significant.

To determine if the replacement of promoters in these strains resulted in increased SMG expression, RT-qPCR was conducted to assess expression levels. Expression in the P*_glnA_2_-ofaABC* strain was compared with wild-type in SMG, which revealed that although transcript levels of *ofaA*, *ofaB*, and *ofaC* were elevated, the increase was statistically significant only for *ofaA* and *ofaB* (Fig. 9A).

For P_BAD_-*ofaABC*, in the absence of inducer, expression of the cluster was nearly undetectable (Fig. 9B). Induction with 0.1% arabinose led to significantly higher transcript levels of *ofaA*, *ofaB*, and *ofaC* compared to the uninduced control. (Fig. 9B). To investigate whether higher inducer concentration could enhance expression, 0.2% arabinose was tested. Although transcript levels of *ofaA*, *ofaB*, and *ofaC* appeared higher than the uninduced control, they did not differ from those observed at 0.1% arabinose (*ofaA* [*P* = 0.294], *ofaB* [*P* = 0.226], and *ofaC* [*P* = 0.209]) (Fig. 9B; Table S4). Interestingly, the higher 0.2% arabinose inducer appeared to lead to lower overall expression than the 0.1% inducer, which is opposite of what would be expected, although given the limited number of biological replicates, this result should be interpreted as a trend rather than a definitive quantitative difference. There is no clear explanation for this result.

In the *B. ambifaria pqq* overexpression strain in the absence of inducer, expression levels of all four genes were identical to WT. Induction using 0.02% rhamnose led to a strong, significant upregulation of *pqqB* at dual cutoffs of >1.5-fold and *P* < 0.05, while *pqqC* and *pqqE* did not exhibit significant changes compared to the uninduced control (Fig. 9C; Table S4). Expression of *pqqD* even had an apparent (but not statistically significant) decrease in expression. These results suggest that while the inducible system can effectively enhance expression of some genes within the operon (e.g., *pqqB*), others may be subject to additional layers of regulation or differential transcript stability. While these results may suggest that induction will not necessarily result in increased PQQ production, the *pqqB* gene has been implicated to play a key role in pyrroloquinoline quinone synthesis and transport across the cytoplasmic membrane into the periplasm, important steps linked to its role in plant growth promotion (63). Also, studies have shown that *pqqB* exhibited the highest expression under conditions where pyrroquinoline levels were maximized, suggesting that expression of *pqqB* is significantly correlated with metabolite accumulation (64). Therefore, the observed upregulation of *pqqB* following induction may still enhance pyrroquinoline production, even if full operon induction is not uniformly achieved.

### Promoter replacement reduced orfamide A production but enhanced pyrroquinolone production

**Fig 10:**
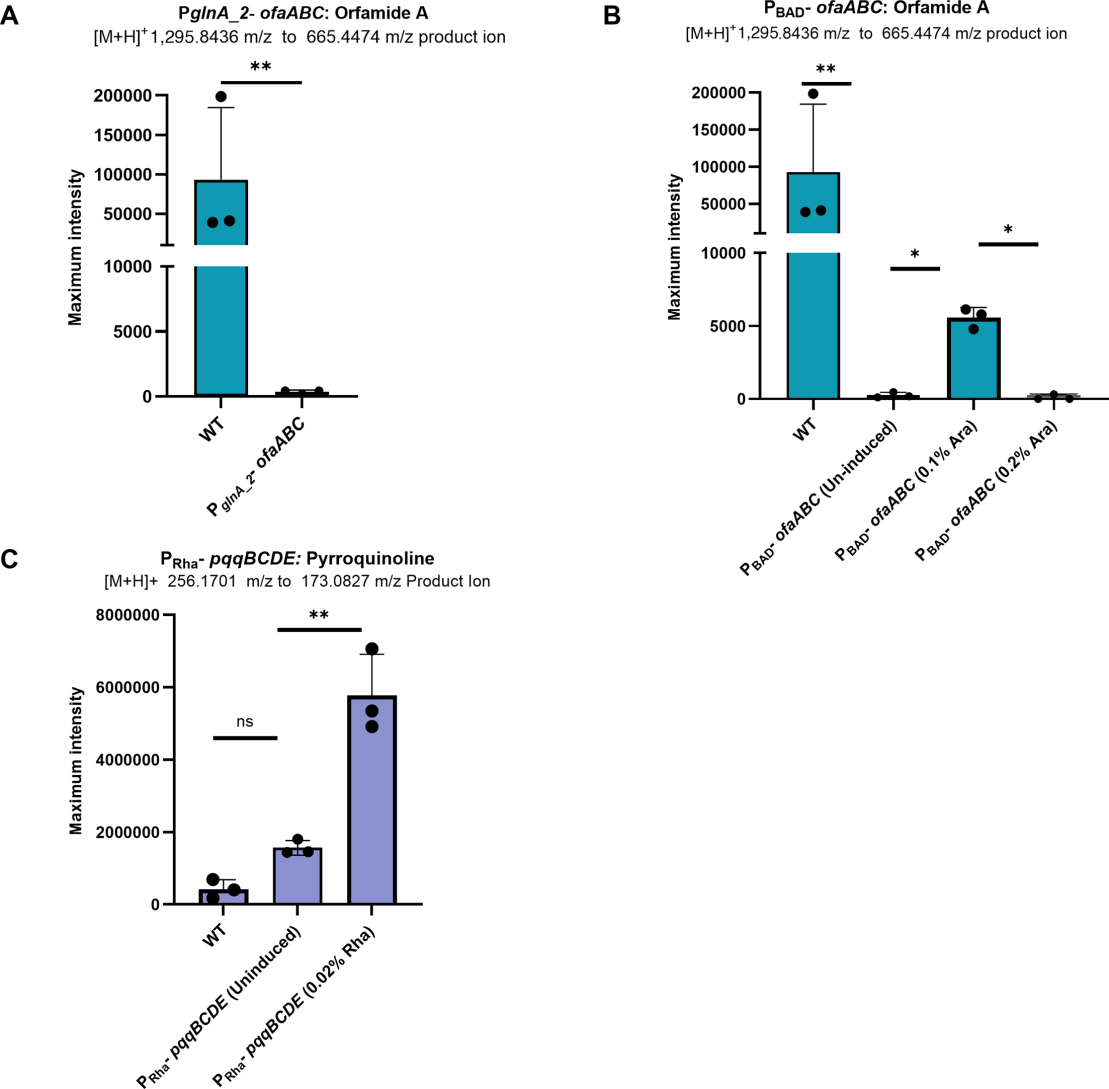
Targeted metabolite analysis of engineered strains under SMG conditions. (**A**) Orfamide A production in WT and P*_glnA_2_-ofaABC*; the promoter-replaced strain showed a significant reduction. (**B**) Orfamide A levels in WT and P_BAD_-*ofaABC* with and without arabinose induction (0.1% and 0.2%). Induction with 0.1% arabinose showed a significant increase in orfamide A production over uninduced, but less than WT levels, while almost no orfamide A was detected at 0.2% induction. (**C**) PQQ production in P*_rha_-pqqBCDE* comparing uninduced and 0.02% rhamnose-induced conditions. Induction resulted in a significant increase in metabolite detection. Error bars represent standard deviation from three biological replicates. Statistical significance was determined using a two-tailed Welch’s *t*-test; *P* < 0.05 (*), *P* < 0.01 (**), ns = not significant.

Engineered strains from the above section were analyzed by targeted metabolomics analysis for the metabolites of interest. Orfamide A production was monitored via the precursor ion at 1,295.8436 *m/z* and its fragment ion at 665.4474 *m/z*, while quinolone was detected by monitoring the precursor ion at 256.1701 *m/z* and the fragment ion at 173.0827 *m/z*, as described previously. Surprisingly, LCMS monitoring of orfamide A in P_glnA-2_*-orfABC* revealed almost no detection (Fig. 10A), suggesting strong transcription of this operon inhibits orfamide A synthesis. In the P_BAD_-*ofaABC* strain, orfamide A was nearly undetectable in the uninduced strain (Fig. 10B), while induction with 0.1% arabinose led to a statistically significant increase in orfamide production. However, the overall metabolite concentration remained lower than that of the wild-type strain. Induction at the higher 0.2% arabinose not only further enhanced orfamide levels but also yielded metabolite concentrations comparable to the uninduced state. These results, combined with the P*_glnA-2_-ofaABC* results, suggest that the more the *ofaABC* operon is induced, the less orfamide A is produced. In contrast, in the P_Rha_*-pqqBCDE* strain with no induction, PQQ detection was comparable to WT, but induction with 0.02% L-rhamnose led to >10-fold elevated quinolone levels (Fig. 10C). Therefore, while the full operon was not uniformly induced, the strong expression of *pqqB* appears to be sufficient to produce higher levels of PQQ.

## DISCUSSION

Microgravity imposes unique physiological stresses on bacteria, altering their behavior and metabolism in ways that can affect the biological systems they interact with, including plants and humans. As space missions extend and bacterial communities play increasing roles in ship systems, understanding how microgravity impacts bacterial secondary metabolite production is essential. Previous studies in this area have primarily focused on human health and industrially relevant species such as *Streptomyces*, *Escherichia coli*, and *Bacillus* (20). These studies have shown that microgravity can alter secondary metabolite production, but the specific outcomes vary depending on the bacterial species and compound. For instance, simulated microgravity resulted in a significant reduction in β-lactam antibiotic (cephamycin C) and rapamycin production by *Streptomyces* species (65), with rapamycin levels decreasing by up to 90% (66), whereas the production of Gramicidin S by *Bacillus brevis* was unaffected (67). In contrast, spaceflight exposure led to a 13%–18% increase in the production of Nikkomycins X and Z, antifungal compounds produced by *Streptomyces ansochromogenus* (17, 68). There remains a critical knowledge gap in understanding how plant-beneficial bacteria alter their metabolite profiles in space. Addressing this gap is vital not only for a fundamental understanding of secondary metabolite production but also for developing biocontrol agents that can prevent fungal contamination that can cause outbreaks in space-based agricultural systems. This study provides foundational insights into how different genetic engineering strategies can be used to manipulate biocontrol strains, influencing their biosynthetic pathways. These findings advance our understanding toward a critical first step toward future efforts aimed at optimizing bacterial performance to enhance plant defense and growth in space-based agricultural systems.

In this study, both organisms exhibited a delayed exponential phase growth compared to normal shaking, suggesting slower adjustment to physiological stress during early growth within the clinostat. Additionally, both organisms displayed reduced biomass compared to the shaking environment. This observed reduction is likely due to the combination of reduced oxygenation and absence of convection-driven mixing relative to the cells under SMG, creating a quiescent fluid environment affecting membrane transport, which restricts nutrient distribution and leads to localized nutrient depletion and prolonged growth kinetics of cells. These changes represent the principal physiological stressors of a microgravity-like environment, distinct from the mechanical stress encountered in traditional shaking or static cultures. While the experimental setup (vertical vs. horizontal orientation) attempts to isolate the gravity vector as the major difference between experiment and control scenarios, it is possible that variation in oxygenation of cultures inherent to the clinostat, or other vessel-dependent variables, may also contribute to observed differences. These altered growth patterns can result in, or be the result of, large-scale effects on gene expression and metabolite production (69), which can be detrimental for developing reliable biocontrol agents. Transcriptomic analysis in this study provided insights into altered regulation of functional categories related to primary metabolism, including carbohydrate, lipid, and amino acid transport and metabolism, as well as biofilm formation and post-translational modifications. These fundamental pathways are foundational to cellular growth and energy balance, and their altered regulation suggests that microgravity imposes a broad metabolic stress that disrupts core functions.

Based on these results, simulated microgravity was found to distinctly impact bacterial secondary metabolism over time, with broader transcriptional suppression observed at later stages. Under normal gravity, secondary metabolites are typically produced during or after the late exponential growth phase. Therefore, the relatively higher yields observed under simulated microgravity at early time points (e.g., Day 1) likely represent an acute stress response rather than a true adaptive shift. While these early-phase differences were statistically significant, the considerable variability between replicates makes it challenging to draw firm conclusions about their biological relevance. This initial surge may result from immediate environmental stressors, such as low shear force or nutrient limitations, rather than transcriptional regulation alone. Because effective biocontrol requires metabolites to be maintained over the duration of pathogen exposure (70, 71), later time points may provide a more relevant measure of sustained biosynthetic capacity under SMG conditions. As such, Day 3 provides a more appropriate reference point for evaluating sustained transcriptional and metabolic responses, minimizing the confounding effects associated with early-phase stress responses. Therefore, in this study, all downstream analyses were focused on changes observed at Day 3.

Targeted analysis of biosynthetic clusters revealed differential expression patterns for genes involved in the production of pyoluteorin, 2,4-DAPG, and orfamide A in *P. protegens*, as well as pyrroquinoline in *B. ambifaria*. In particular, the *ofaABC* cluster in *P. protegens* and the *pqqBCDE* operon in *B. ambifaria* were both significantly downregulated after 72 h of simulated microgravity exposure, indicating a possible shift in metabolic priorities toward core cellular functions during prolonged stress. Investigation of altered gene expression patterns revealed a correlation between gene expression and production output. Among all metabolites, orfamide A showed the strongest correlation, with increased yield aligning with *ofaABC* upregulation at Day 1 and significant downregulation at Day 3 corresponding to reduced compound level. This temporal switch suggests an early stress-induced transcriptional activation followed by suppression, likely due to energetic constraints or feedback inhibition in prolonged microgravity exposure. Similar to orfamide A, *B. ambifaria* quinolone production also followed a co-related biphasic trend, with higher levels under SMG on Day 1 but significantly reduced yields at Day 3. Such regulation is consistent with previous spaceflight studies, where the production of secondary metabolites like actinomycin D by *Streptomyces plicatus* increased during early time points in space but declined with prolonged exposure, highlighting the importance of stress duration in determining biosynthetic outcomes (18, 19). Similarly, cyanobacteria such as *Microcystis aeruginosa* showed elevated microcystin production and nitrogen uptake under simulated microgravity in the early phase, potentially reflecting a metabolic response to energy demands and light stress (72). Conversely, some results did not correlate well; notably, 2,4-DAPG production remained consistently lower under SMG at both time points, despite mixed transcriptional signals from the *phl* gene cluster. This lack of coordinated transcription, coupled with uniformly reduced 2,4-DAPG levels, suggests that its biosynthesis may be more susceptible to post-transcriptional regulation or limited by precursor availability, potentially due to alterations in primary metabolism under SMG. However, for pyoluteorin, the trend was intermediate. Most *plt* genes were upregulated on Day 1, consistent with observed increased pyoluteorin production, but by Day 3, metabolite levels declined despite elevated transcript levels, further suggesting affected translation efficiency, or associated regulatory networks, or metabolite stability under SMG. Thus, the observed time-dependent shifts in gene expression and metabolite production, along with instances of transcription-metabolite decoupling, show the complexity of impacted secondary metabolism in microgravity. These insights are critical for developing robust, reliable microbial strains for space agriculture, where consistency in biocontrol activity and metabolite output will be essential.

As observed from this study, downregulated transcriptional networks and suppressed production of key plant-beneficial compounds may influence bacterial-assisted plant growth in space environments; however, these potential effects require validation through experiments that directly assess plant-microbe interactions. While applied genetic engineering strategies are well-characterized in *Pseudomonas* (44, 49, 73, 74), the transcriptional regulation approach applied here, based on strong promoter replacement, was chosen for its relative simplicity and minimal disruption to broader cellular networks. However, this strategy alone appeared insufficient, at least for orfamide A. These results are especially confusing. When the native promoter was replaced with the inducible P_BAD_ promoter, no expression or metabolites were seen in the absence of induction, which is expected. Induction with 0.1% arabinose caused transcript levels of *ofaA* to reach levels comparable to the wild-type (Fig. 9B); however, orfamide A production remained significantly lower, reaching only a fraction of the WT levels (Fig. 10B). Induction at the higher 0.2% arabinose led to a reduction in transcript levels compared to 0.1% arabinose (Fig. 9B), and essentially no detectable orfamide A (Fig. 10B). It is counterintuitive that an increased concentration of inducer could lead to a decreased level of transcript, but this phenomenon was previously observed in tightly regulated inducible promoters (75, 76), possibly due to regulatory saturation or feedback inhibition that suppress expression at higher inducer levels. Here, at least the trends of transcript production matched the trends in metabolite production. However, this trend did not hold when the native promoter was replaced with the P_glnA_2_ promoter, where the transcript levels were roughly three times higher than WT (Fig. 9A), but essentially no orfamide A was produced (Fig. 10A). The lack of orfamide A production despite elevated P_glnA_2_-driven transcripts could, in principle, reflect translational limitations or altered transcript context. The native ribosomal binding site was retained; hence, the discrepancy is unlikely due to the removal of translation signals. However, promoter replacement can affect mRNA structure, translational efficiency, or operon-level regulation, highlighting the importance of post-transcriptional and metabolic constraints in biosynthetic gene clusters such as *ofaABC*. In general, the stronger the induction of the *ofaABC* promoter, the less orfamide A is produced, but only in some cases is this correlated with actual transcript levels. Biosynthetic gene clusters like *ofaABC* are typically regulated through multilayered systems, such as the Gac-Rsm pathway. Activation of the response regulator GacA directly promotes the transcription of small RNAs (RsmX/Y/Z) that lift translational repression, thereby facilitating expression of target biosynthetic genes (77). Therefore, even if a high level of transcript can be achieved, it may not guarantee effective gene expression due to additional post-transcriptional controls. Thus, effective engineering of *ofaABC* requires an integrated strategy that addresses both transcriptional and post-transcriptional regulation. Importantly, manipulating global regulators such as Gac-Rsm may have unintended consequences on other pathways, complicating targeted interventions. As noted, even well-characterized promoters can behave unpredictably when placed in new contexts, highlighting the challenges of rational promoter engineering without iterative optimization (2). These findings suggest that promoter replacement alone may be insufficient to maximize biosynthetic yield. Instead, regulatory fine-tuning may be required, as metabolic output is often tightly linked to promoter strength, gene dosage, and the coordination between primary and secondary metabolism (78). We note that RT-qPCR experiments were limited in biological replicates due to the constraints of clinostat-based cultivation. While each replicate was measured in technical duplicates, which ensures consistency, these technical replicates do not replace true biological replication. These results, therefore, indicate trends and directional changes rather than definitive quantitative conclusions.

While promoter replacement proved insufficient at substantially increasing expression, plasmid-based overexpression of the *pqqBCDE* operon in *B. ambifaria* resulted in significantly increased transcript levels (Fig. 9C) and higher metabolite production under 0.02% rhamnose induction (Fig. 10C). The choice of a plasmid-based system in this organism was based on multiple possible constraints associated with *Burkholderia* genetics: notably, low transformation efficiency, resistance to common antibiotic selection markers, and the incompatibility of standard recombination systems (61, 79, 80). Furthermore, the *pqqBCDE* operon is embedded in a complex regulatory landscape, influenced by quorum sensing and environmental signals. Similar regulatory intricacy has been reported for quinolone derivative biosynthetic clusters in *B. ambifaria*, such as the *hmqABCDEFG* operon, which is governed by multiple quorum-sensing systems and additional layers of control (62). In such contexts, chromosomal modifications risk unintended interference with neighboring genes or global transcriptional regulators. Plasmid-based systems, by contrast, offer modularity and tunable expression with minimal disruption to native regulation. What is not clear is if the successful increase in pyrroloquinoline production is due to the removal of the operon from the genomic and native promoter context or if it is because this particular metabolic pathway is less constrained by post-transcriptional or metabolic regulation. Regardless, these results further emphasize the variability of microbial responses, not just to SMG but to genetic engineering. It is unlikely to be a single universal bacterial response or single methodology to alter those responses. Understanding and manipulating bacterial traits to provide human benefits appears to require much more direct hands-on experimentation.

Due to the coordination required across the experimental timeline and the limited availability of clinostat vessels, the number of biological replicates was necessarily restricted. Three replicates were selected in accordance with previous transcriptomic (81, 82) and metabolomic (83, 84) studies, providing sufficient power to capture reproducible trends. Additional replicates would further enhance the robustness of these findings, particularly under non-traditional experimental conditions such as simulated microgravity.

Overall, this study bridges fundamental insights into bacterial secondary metabolism with the applied challenges of space agriculture. Our findings demonstrate that microgravity profoundly impacts gene expression and metabolite production in plant-beneficial bacteria, highlighting the need for a broader understanding of how to strategically select and optimize biocontrol agents for space environments. While the molecular alterations may have implications for plant-microbe and microbe-pathogen interactions, such functional effects were not directly assessed here. Future studies integrating plant interaction assays and co-culturing will be important to determine whether these molecular responses translate into altered biocontrol activity or plant growth outcomes under spaceflight conditions. Developing robust microbial systems for space applications will require a systems-level understanding of stress responses, nutrient dynamics, and temporal regulation. Nevertheless, this work lays the groundwork for engineering reliable biocontrol strains through context-aware genetic strategies tailored to the microgravity condition of spaceflight.

## MATERIALS AND METHODS

### Bacterial strains, media, hardware, and simulated microgravity growth conditions

*P. protegens* Pf-5 and *B. ambifaria* AMMD were used as the primary strains in this study. *E. coli* DH5α was employed for cloning and plasmid propagation, while *E. coli* S17 was used as the donor strain for conjugation. *P. protegens* was cultured in King’s B (KB) media (20 g/L peptone, 5 g/L glycerol, 1.5 g/L KH_2_PO_4,_ 5 mM MgSO_4_, and 1.5% agar for plates) at 30°C. *E. coli* DH5α and S17 were grown in LB media (10 g/L tryptone, 5 g/L yeast extract, 2.5 g/L NaCl, and 1.5% agar for plates) at 37°C. *B. ambifaria* was cultured in Basal Salt Media-G (BSM-G) containing 4% glycerol (4.25 g/L K_2_HPO_4_.3H_2_0, 1.00 g/L NaH_2_PO_4_.H_2_0, 2.00 g/L NH_4_Cl, 0.20 g/L MgSO_4_.7H_2_0, 0.012 g FeSO_4_.7H_2_0, 0.003 g/L MnSO_4_.H_2_0, 0.003 g/L ZnSO_4_.7H_2_0, 0.001 g/L CoSO_4_.7H_2_O, and 0.10 g/L nitrilotriacetic acid) (85). Antibiotics were supplemented as needed: kanamycin 50 µg/mL (for *E. coli* DH5α and S17), tetracycline 12 µg/mL (for *E. coli*, 200 µg/mL for *P. protegens*), and trimethoprim (50 µg/mL for *E. coli* and 100 µg/mL for *B. ambifaria*). Arabinose (0.1% and 0.2%) (73, 86, 87) and Rhamnose (0.02%) (60, 88) were used as inducers and added to *P. protegens* and *B. ambifaria* cultures as required. All strains and plasmids used in this study are listed in Tables S6 and S7.

Both *P. protegens* and *B. ambifaria* were cultured using a clinostat ground-based microgravity simulation system (Synthecon). Cultures were injected into HARVs using syringes. Cultures rotated in a horizontal orientation were exposed to simulated microgravity and served as experimental samples, while control cultures were rotated vertically to provide traditional gravity. To maintain the effect of microgravity, the liquid-filled vessels were rotated at a constant speed, continuously reorienting the gravity vector. The rotation speed and vessel radius were optimized to keep cells in suspension and prevent sedimentation or centrifugation by balancing gravitational, centrifugal, and Brownian motion forces (48). With a diameter of the vessel or culture less than 5 cm, rotating a clinostat at or below ω= 0.5 rad/s or 10 rpm will maintain microgravity simulation below g'=10^−3^ (41, 89). Thus, a 5 mL vessel with a 1 cm radius rotated at 4.8 rpm provided ideal parameters for achieving a reduced gravity range of 10⁻³–10⁻⁶ g for this study. Aeration for strictly aerobic cultures was maintained via a semipermeable membrane at the back of the HARVs. All cultures were grown in triplicate, with one condition/orientation (treatment or control) processed at a time using the Rotary Cell Culture System. The growth rate of *P. protegens* and *B. ambifaria* was monitored at both orientations by measuring OD₆₀₀ in duplicate, using a Nanodrop spectrophotometer (Thermo Fisher Scientific) at 4-h intervals over a 72-h period. To avoid introducing air bubbles, which could disrupt the simulated microgravity environment, 5 µL of culture was carefully withdrawn and replaced with 5 µL of fresh medium. This sampling approach ensured minimal system disturbance and prevented dilution artifacts. For downstream assays described in the following sections, cultures were terminated, and samples were collected on Day 1 and Day 3 under both simulated microgravity (SMG) and traditional gravity (TG) conditions.

### Strain construction

All plasmids, primer sequences, and promoter sequences used in the study are listed in Tables S7 to S9, respectively. For creating *lacZ* fusion strains of *P. protegens* for β-galactosidase reporter assays, promoter sequences of *rpoD, rpsA, oprf*, and *glnA_2* were amplified and fused with a promoterless *lacZ* gene present in BgIII and KpnI digested pMP220 (Addgene) to generate pGS004 (P*_dnaG_-lacZ*), pGS005 (P*_rpsA_- lacZ*), pGS006 (P*_rrA_-lacZ*), and pGS007 (P*_glnA_2_-lacZ*) plasmid constructs using Gibson Assembly Mastermix (New England Biolabs). The ribosomal binding site for *lacZ* translation is vector-derived from pMP220; the cloned promoter fragments contained only transcriptional regulatory sequences. These cloned constructs were introduced into *E. coli* DH5α by chemical transformation (90), and plasmids were inserted into *P. protegens* cells via electroporation (91) to generate *P. protegens* PC-0680, PC-0681, PC-0682, and PC-0683 strains for β-galactosidase reporter assay.

Allelic replacement was used for replacing the native *ofaABC* promoter with either the arabinose-inducible P_araBAD_ (P_BAD_) from *E. coli* (86) or the strong native endogenous P*_glnA_2_* of *P. protegens*. To generate the promoter replacement strains in *P. protegens*, an 892 bp fragment upstream and an 889 bp fragment downstream of P*_ofaABC_* were PCR amplified using primers ofaAupF/ofaAupR and ofaAdnF/ofaAdnR, respectively. A 244 bp P*_glnA_2_* fragment was amplified using P*_glnA_2_*F/P*_glnA_2_*R from *P. protegens* genomic DNA, and a 227 bp P_BAD_ fragment was amplified from the pBADTrfp plasmid using primers P_BAD_F/P_BAD_R. The upstream and downstream fragments were then cloned with a middle promoter fragment into HinDIII and EcoRI-digested pEX18Tc (91, 92) using Gibson Assembly Mastermix to create plasmids pGS001 (P*_glnA_2_*) and plasmid pGS002 (P_BAD_).

Plasmids pGS001 and pGS002 were transformed by electroporation into the mobilizing strain *E. coli* S17 (92). These constructs were subsequently introduced into *P. protegens* by biparental conjugation to generate the chromosomal promoter replacement strains and P*_glnA_2_-ofaABC* and P_BAD_-*ofaABC*, respectively. Briefly, biparental matings were performed between *E. coli* S17 harboring either pGS001 or pGS002 and *P. protegens* on LB agar containing 1% (vol/vol) glycerol (93). Transconjugant *P. protegens* colonies were selected on King’s B agar media containing 200 µg/mL tetracycline and 100 µg/mL streptomycin (innate resistance of *P. protegens*). Surviving colonies were grown without selection in LB broth at 30°C overnight with shaking at 180 rpm and plated on 10% sucrose LB agar for counter-selection against *sacB*-carrying cells. Sucrose-resistant colonies were patched in parallel onto LB agar containing 10% sucrose and King’s B agar media with 200 µg/mL tetracycline to further confirm the absence of the pEX18Tc vector backbone. Tetracycline-sensitive colonies were screened via confirmation PCR using primers flanking the *ofaABC* promoter region to identify recombinants with altered amplicon sizes relative to the parental strain. Promoter replacement was further verified by sequencing.

To generate the *pqqBCDE* overexpression strain of *B. ambifaria*, the *pqqBCDE* operon was PCR-amplified from *B. ambifaria* genomic DNA using pqqBCDE-F/pqqBCDE-R primers. This amplified fragment was cloned into Apal and PstI digested pSCrhaB2, a replicating plasmid with a rhamnose-inducible promoter (Addgene) (88) using Gibson Assembly Mastermix, resulting in plasmid pGS003, in which the operon is placed under the control of rhamnose-inducible promoter (P_Rha_). The plasmid was then inserted into *B. ambifaria* via electroporation (79) to generate the overexpression strain P_Rha_-*pqqBCDE*

### RNA-seq analysis

Total RNA was extracted from cultures of *P. protegens* and *B. ambifaria* at 24 h (Day 1) and 72 h (Day 3) of incubation using RNAprotect “Enzymatic Lysis of Bacteria” protocol (Qiagen), with slight modifications, followed by purification with the PureLink RNA Mini Kit (Invitrogen). Briefly, 1.5 mL of overnight culture was centrifuged at 17,000 × *g* for 2 min at 4°C, and the pellet was resuspended in 500 µL of growth medium. RNAprotect Bacteria Reagent (Qiagen) was added at a 2:1 ratio to stabilize RNA, followed by a 10-min incubation on ice. Cells were pelleted (17,000 × *g*, 5 min, 4°C), and the pellet was resuspended in 200 µL of lysis buffer (TE buffer [10 mM Tris-HCl, 1 mM EDTA, pH 8] containing 10 mg/mL lysozyme), followed by incubation on ice for 10 min with intermittent vortexing (25 s every 2 min) to ensure thorough exposure of cells to the lysis buffer. Lysates were processed using the PureLink RNA Mini Kit according to the manufacturer’s protocol, and RNA was eluted in 50 µL of nuclease-free water. RNA concentration was measured using a NanoDrop spectrophotometer (Thermo Fisher Scientific). All RNA samples were prepared from cultures grown independently in triplicate and submitted for sequencing to the Center for Genomics and Bioinformatics at Indiana University, Bloomington.

RNA integrity was assessed by an Agilent 2100 Bioanalyzer (Agilent Technologies). Enrichment of mRNA was performed by removing rRNA using the MICROBExpress rRNA removal kit (Ambion). Before library preparation, cDNA was synthesized complementary to mRNA using random primers and Reverse Transcriptase. Second strands complementary to newly synthesized strands were synthesized, creating double-stranded DNA from the mRNA template. This DNA was used for library preparation using Nextera XT DNA Library Prep Kit (Illumina), followed by Illumina sequencing analysis. After sequencing, data analysis was performed in-house. Raw reads were assessed using FASTQC (v0.12.1) to evaluate sequencing quality, including checks for adapter contamination, GC content, and per-base sequence quality. To improve data quality, adapter trimming and quality clipping were performed using Trimmomatic (v0.39), a widely used tool that applies adaptive algorithms, such as the sliding window approach, to remove low-quality regions and enhance the reliability of downstream analyses (94, 95). Sickle (v1.33) was used for quality-based trimming to remove low-quality reads, ensuring that only high-quality sequences were retained for downstream analysis and preserving overall data integrity (96). Trimmed reads were aligned to the reference genome using STAR (v2.7.11b), producing alignment files (SAM format) that were subsequently used as input for HTSeq-count (v2.0.5). HTSeq was employed to quantify raw gene expression counts by mapping sequencing reads to annotated genomic features, such as genes (97). Differential gene expression analysis was then performed using the DESeq2 package (v1.38.3), which applies negative binomial distribution modeling to identify statistically significant expression differences between experimental conditions (98). Statistical significance of expression changes between conditions was determined using the Wald test, and genes with an adjusted *P* < 0.05 were considered significantly differentially expressed. For all analyses, three biological replicates were used, with counts from technical replicates averaged prior to analysis. For gene mapping and annotation, reference genomes were obtained from the NCBI: *P. protegens* (Accession No. CP000076) and *B. ambifaria* (Accession No. CP009798.1).

### RT-qPCR analysis

Relative mRNA expression of various strains was assessed by RT-qPCR. Total RNA was extracted from clinostat-grown cultures at 24 and 72 h using the same protocol as for RNA-seq. Two biological replicates with two technical replicates each were used for each analysis. RNA concentrations were measured using a NanoDrop spectrophotometer. To remove genomic DNA contamination, samples were treated with TURBO DNase (2 U/µL) according to the manufacturer’s protocol (Invitrogen). Reverse transcription was performed for cDNA synthesis using the QuantiTect Reverse Transcription Kit (Qiagen). RT-PCR was conducted using gene-specific primers to confirm successful cDNA synthesis. qPCR was then carried out using the QuantiTect SYBR Green Kit (Qiagen) on a Rotor-Gene Q real-time PCR system (Qiagen, software v 2.3.5.1). Gene-specific primers were used, including primer pairs qPCRofaA-F/R for *ofaA,* qPCRofaB-F/R for *ofaB,* and qPCRofaC-F/R for *ofaC,* with rpsA-F/R used for the reference gene *rpsA in P. protegenes*. For *B. ambifaria,* gene-specific primers including qPCRpqqB-F/R for *pqqB,* qPCRpqqC-F/R for *pqqC,* qPCRpqqD-F/R for *pqqD*, and qPCRpqqE-F/R for *pqqE* were used, with rpsL-F/R used for the reference gene *rpsL*. Primers were designed to amplify 180–210 bp regions of each transcript. The qPCR reaction mix contained 1.0 µM of each primer, 25 µL of Absolute SYBR Green qPCR mix, 1 µL of cDNA, and 22 µL of nuclease-free water, resulting in a total reaction volume of 50 µL. Thermal cycling conditions were as follows: initial denaturation at 95°C for 15 min, followed by 40 cycles of 94°C for 30 s, annealing at 64°C (*P. protegens* strains) or 61°C (*B. ambifaria* strains) for 30 s, and extension at 72°C for 30 s. This was followed by a final melt curve stage: 95°C for 1 min, 55°C for 30 s, and 95°C for 30 s. Ct values were normalized using the respective reference genes, and relative gene expression levels were calculated using the 2⁻ΔΔCt method (99). Two technical replicates were averaged within each biological replicate. Differences between conditions were assessed using a two-tailed Welch’s *t*-test, with *P* < 0.05 considered statistically significant.

### LC-MS sample preparation

Metabolite extraction was conducted in three biological replicates per condition at comparable OD₆₀₀ values. Cultures were harvested at 24 h (Day 1) and 72 h (Day 3), followed by centrifugation at 20,000 × *g* for 15 min at 5°C. The supernatants were collected for LC-MS analysis, while the cell pellets were reserved for RNA extraction. Samples were prepared in triplicate. The collected supernatants (3 mL each) were subjected to solid-phase extraction using 100 mg C18 reversed-phase SPE cartridges for primary purification. Supernatants were passed through SPE columns, washed with 1,000 µL of Milli-Q H₂O, and eluted with 1,000 µL of 100% acetonitrile (MeCN). The eluted samples were further centrifuged to remove any residual turbidity, ensuring sample clarity prior to LC-MS analysis. Finally, the purified MeCN fractions were analyzed by LC-MS to quantify the targeted metabolites. LC-MS analysis of *P. protegens* metabolites, including pyoluteorin, 2,4-diacetylphloroglucinol, and orfamide A, was performed using an Agilent 6530C Q-TOF LC/MS system, which is equipped with an Agilent Jet Stream source and a 1260 Infinity II pump stack. Separation was achieved using a Kinetex EVO C18 column (2.6 μm, 50 × 2.1 mm, 100 Å; Phenomenex) with a mobile phase of 0.1% formic acid in Milli-Q H₂O (solvent A) and 0.1% formic acid in MeCN (solvent B). The gradient program was as follows: initial elution at 10% B for 3 min, followed by a linear gradient to 25% B by 5 min, then a linear increase to 99% B over 7.5 min, held at 99% B for 5 min, followed by a return to initial conditions over 1.5 min and a re-equilibration phase for 3 min. Targeted MS/MS acquisition was performed at *m/z* 271.9876 for pyoluteorin (RT 6.83 min, ΔRT 1.0, CE 25, fragment mass at 137.0225 *m/z*), *m/z* 211.0601 for 2,4-DAPG (RT 7.72 min, ΔRT 1.0, CE 25, fragment mass at 193.0483 *m/z*), and *m/z* 1,295.8436 for orfamide A (RT 11.63 min, ΔRT 1.0, CE 53.89, fragment mass at 665.4474 *m/z*). The flow rate was set at 450 μL/min with a 10 μL injection volume. The MS run was divided into three segments: 0–3 min (flow to waste), 3–19.5 min (flow to MS), and post-19.5 min (flow to waste). A similar LC-MS method was applied for the analysis of quinoline derivatives produced by *B. ambifaria*, with slight modifications to the gradient program. The gradient was as follows: initial elution at 10% B for 3 min, followed by a linear gradient to 25% B for 5 min, then a linear increase to 99% B over 7.5 min, held at 99% B for 3 min, followed by a return to initial conditions over 2 min, and a re-equilibration phase for 2.5 min. Targeted MS/MS acquisition was conducted at *m/z* 256.1701, corresponding to hydroxyquinoline, with a retention time of 8.24 min (ΔRT = 0.75 min) and monitoring the fragment ion at 173.0827 *m/z* (CE 35.29). The MS run was divided into three segments: 0–3 min (flow to waste), 3–17.5 min (flow to MS), and post-17.5 min (flow to waste). Data were acquired in positive ion mode and centroid mode. Source conditions were as follows: drying/sheath gas at 325°C/400°C, flows at 10/12 L/min, nebulizer at 50 psi, capillary voltage at 4,000 V, and nozzle voltage at 0 V. Ion optics settings included a fragmentor at 200 V, skimmer at 65 V, and octopole RF at 750 V. Reference mass correction off Agilent’s standard reference mix (*m/z* 922.0098, 121.0509, ±100 ppm) was used throughout the run. Relative metabolite quantification was based on peak area integration, normalized to culture OD₆₀₀ at the time of harvest. The indicated fragment ion of each metabolite was used for relative quantification. Three biological replicates were analyzed per condition, and statistical significance between conditions was assessed using a two-tailed Welch’s *t*-test, with *P*-values < 0.05 considered significant. Data visualization and statistical analysis were conducted using GraphPad software (v10) to generate bar graphs for each metabolite.

### Identification of transcription start sites by 5′ RACE

5′ RACE was performed to identify the transcription start site (TSS) of target genes using the 5′ RACE System for Rapid Amplification of cDNA Ends, Version 2.0 (Invitrogen), with slight modifications to the manufacturer’s protocol. Total RNA was isolated from *P. protegens* cultures grown to mid-exponential phase, using the same extraction method described for RNA-seq and RT-qPCR analyses. Genomic DNA was removed using TURBO DNase treatment, following the protocol specified previously. Gene-specific primers (GSPs) were designed to anneal downstream of the annotated start codons, targeting either the gene of interest or the first gene of the operon in which the gene is present (Table S3). First-strand cDNA synthesis was performed using AMV Reverse Transcriptase (Invitrogen) and GSP1 primers for each target gene. The GSP1 primer used included *rpsA*-GSP1, *glnA*_2-GSP1, and *dnaG*-GSP1 for *rpoD* and *rrA*-GSP1 for *oprF*. These primers were designed to synthesize cDNA covering the regions upstream of the start codon, encompassing the transcription start sites of the *rpsA*, *glnA_2*, *dnaG*, and *rrA* genes. Each 20 µL reaction contained 2 µL of 10× AMV buffer, 1 µL of 10 mM dNTP mix, 0.2 µL of Murine RNase inhibitor (NEB), 1 µL of AMV Reverse Transcriptase (Invitrogen), and 1–2 µg of DNase-treated RNA. The final volume was adjusted using DEPC-treated water (Invitrogen). The reaction was incubated at 58°C for 15 min, followed by enzyme inactivation at 70°C for 10 min. To remove residual RNA, the resulting cDNA was treated with RNase Cocktail Enzyme Mix (Invitrogen). Specifically, 1 µL of RNase was added to the 20 µL cDNA reaction, gently mixed, and incubated at 37°C for 30 min.

Poly(dC) tails were added to RNase-treated cDNA using terminal deoxynucleotidyl transferase (TdT; Thermo Scientific). The 20 µL poly(dC) tailing reaction included 4 µL of TdT reaction buffer, 1 µL of 10 mM dCTPs, 1 µL of TdT enzyme, 1 µg of cDNA, and DEPC-treated water to adjust the final volume. The reaction was incubated at 37°C for 15 min, followed by enzyme inactivation at 70°C for 10 min. The resulting dC-tailed cDNA was subjected to first-round PCR amplification using Taq DNA polymerase (NEB). Amplification was performed with a second gene-specific primer (GSP2), located downstream of GSP1, and an abridged anchor primer (AAP). The diluted product from the first-round PCR was used as the template for nested (second-round) amplification. In this step, a third gene-specific primer (GSP3) and the abridged universal anchor primer (AUAP) were used. PCR products from the nested reaction were purified and re-amplified using the same GSP3 and AUAP primers to confirm specificity. The final PCR products were then cloned into a TA cloning vector using T4 DNA ligase. Ligation was carried out at room temperature for 4 h. Cloned fragments were subsequently sequenced to determine the transcriptional start sites.

### β-galactosidase reporter assays

β-galactosidase assays were performed following previously established protocols (56). Briefly, reporter strains were cultured at 30°C for 72 h in the clinostat. All OD₆₀₀ measurements were performed using a NanoDrop spectrophotometer. *P. protegens* strain carrying the empty vector pMP220 was used as the negative control for promoter activity. A 500 µL bacterial culture was mixed with 500 µL of Z-buffer (60 mM Na₂HPO₄·7H₂O, 40 mM NaH₂PO₄·H₂O, 10 mM KCl, 1 mM MgSO₄·7H₂O, pH 7.0). Diluted cells were permeabilized by adding 3–4 drops of toluene, followed by mixing by vortexing. Toluene was evaporated by gentle (90 rpm) shaking at 37°C for 40 min. Samples were equilibrated at 28°C for 5 min before initiating the reaction with 200 µL of cold ONPG (4 mg/mL), and the start time was recorded. Samples were incubated at room temperature until a yellow color developed; the reaction was then stopped by adding 500 µL of 1 M Na₂CO₃, and the stop time was recorded. The optical densities at 420 nm and 550 nm were measured for each sample, where OD₅₅₀ accounts for light scattering caused by cell debris. The corrected OD₄₂₀ value was calculated by subtracting the optical density at 550 nm, thereby minimizing interference from light scattering due to cellular debris, and β-galactosidase activity was quantified using the Miller method to calculate Miller units (100). Each assay was performed in triplicate from three independent cultures (*n* = 9), and the results are reported as mean ± standard deviation. Differences in β-galactosidase activity between conditions or strains were assessed using a two-tailed Welch’s *t*-test, and *P* values < 0.05 were considered statistically significant. Graphs were generated using GraphPad Prism v10.

## Data Availability

The RNA-Seq data set generated during this study is available in NCBI|NLM|NIH under BioProject accession number PRJNA1284919. The link below includes all the RNA-seq data for both the experimental and control samples used in this study, which is now publicly available in http://www.ncbi.nlm.nih.gov/bioproject/1284919. The LC-MS data generated in this study are available in the MassIVE repository under the number MSV000098671. The link below includes all the LC-MS files for both wild type and mutant strains used in this study, which is now publicly available. ftp://massive-ftp.ucsd.edu/v10/MSV000098671/

## SUPPLEMENTAL MATERIAL

Fig. S1A spectrum.02239-25-s0001.docx Shown are the extracted ion chromatograms for 2,4-DAPG (m/z 211.0601), pyoluteorin (m/z 271.9876), and orfamide A (m/z 1295.8436) in Day 1 samples.

Fig. S1B spectrum.02239-25-s0002.docx Shown are the extracted ion chromatograms for 2,4-DAPG (m/z 211.0601), pyoluteorin (m/z 271.9876), and orfamide A (m/z 1295.8436) in Day 3 samples.

Fig. S2 spectrum.02239-25-s0003.docx Shown are the extracted ion chromatograms for pyrroquinoline (m/z 256.1701) in Day 1 and Day 3 samples.

Table S1 spectrum.02239-25-s0004.xlsx The metabolite yield of both wild-type and mutant strains of *Pseudomonas protegens* Pf-5 and *Burkholderia ambifaria* AMMD was quantified under simulated microgravity and normal gravity conditions using LC-MS/MS analysis.

Table S2 spectrum.02239-25-s0005.xlsx Differentially expressed genes at a cutoff of P < 0.05 and fold change of >1.5 at both Day 1 and Day 3 for *P. protegens* RNAseq data*.*

Table S3 spectrum.02239-25-s0006.xlsx Differentially expressed genes at a cutoff of P < 0.05 and fold change of >1.5 at both Day 1 and Day 3 for *B. ambifaria*-RNAseq data.

Table S4 spectrum.02239-25-s0007.xlsx RT-qPCR generated Ct values (2 technical replicates) for 2 biological replicates along with calculated 2-ΔΔCT between SMG and TG at Day 3.

Table S5 spectrum.02239-25-s0008.xlsx RPKM values for each gene under SMG (Replicate 1, 2, and 3) and the list of genes that are highly expressed under SMG for *P. protegens*.

Supplemental tables spectrum.02239-25-s0009.docx Tables S6 to S9.

ASM does not own the copyrights to Supplemental Material that may be linked to, or accessed through, an article. The authors have granted ASM a non-exclusive, world-wide license to publish the Supplemental Material files. Please contact the corresponding author directly for reuse.
