# Supplementary material for: Microgravity effects on secondary metabolism of plant-affecting bacteria
Source: Microbiol Spectr. 2026 Mar 30;14(5):e02239-25. doi: 10.1128/spectrum.02239-25 (PMC13141840; doi:10.1128/spectrum.02239-25)
Supplement: Fig. S1B — Shown are the extracted ion chromatograms for 2,4-DAPG (m/z 211.0601), pyoluteorin (m/z 271.9876), and orfamide A (m/z 1295.8436) in Day 3 samples. [file spectrum.02239-25-s0002.docx]

**DAPG (211.0601 m/z) day 03**


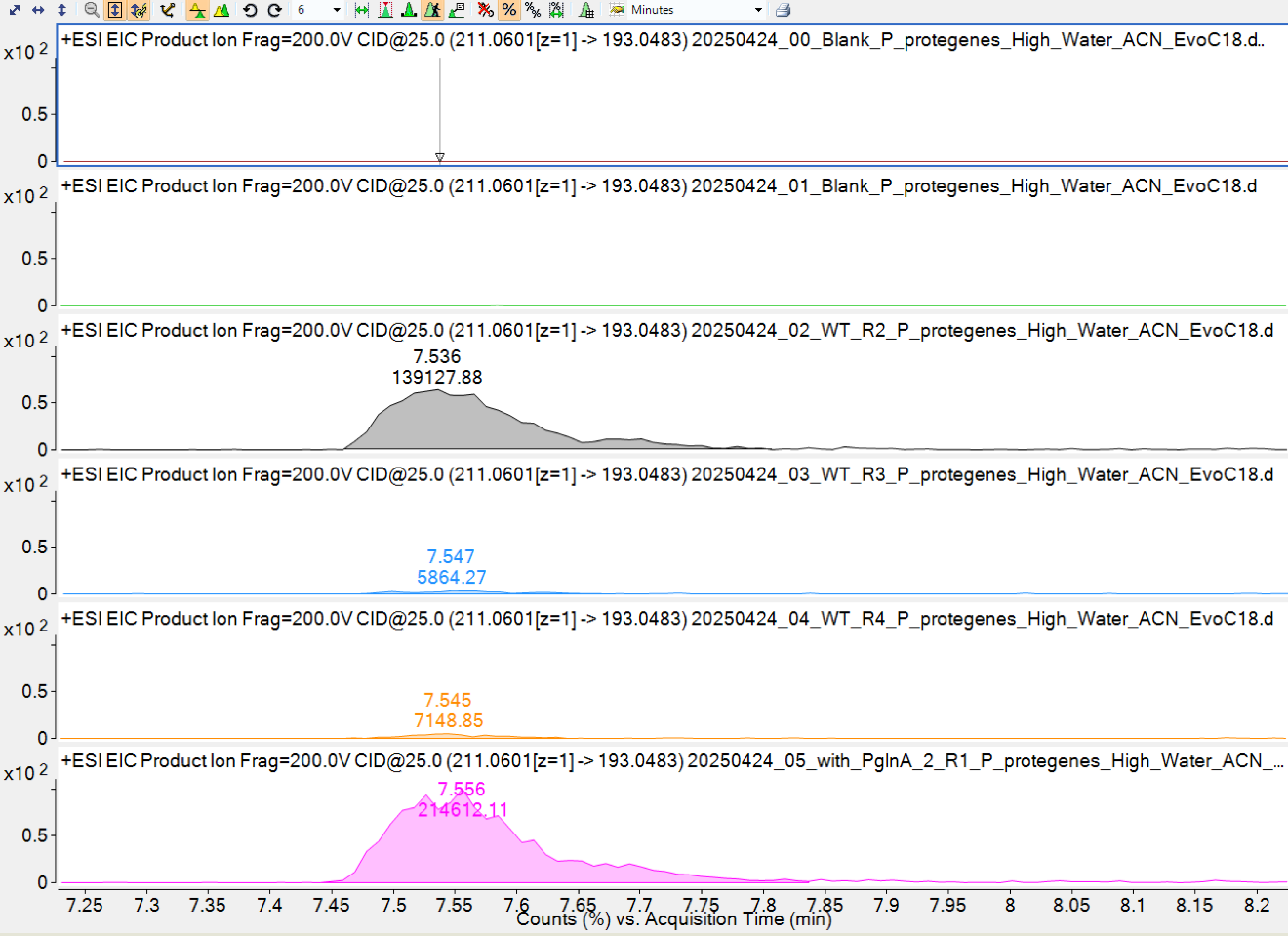


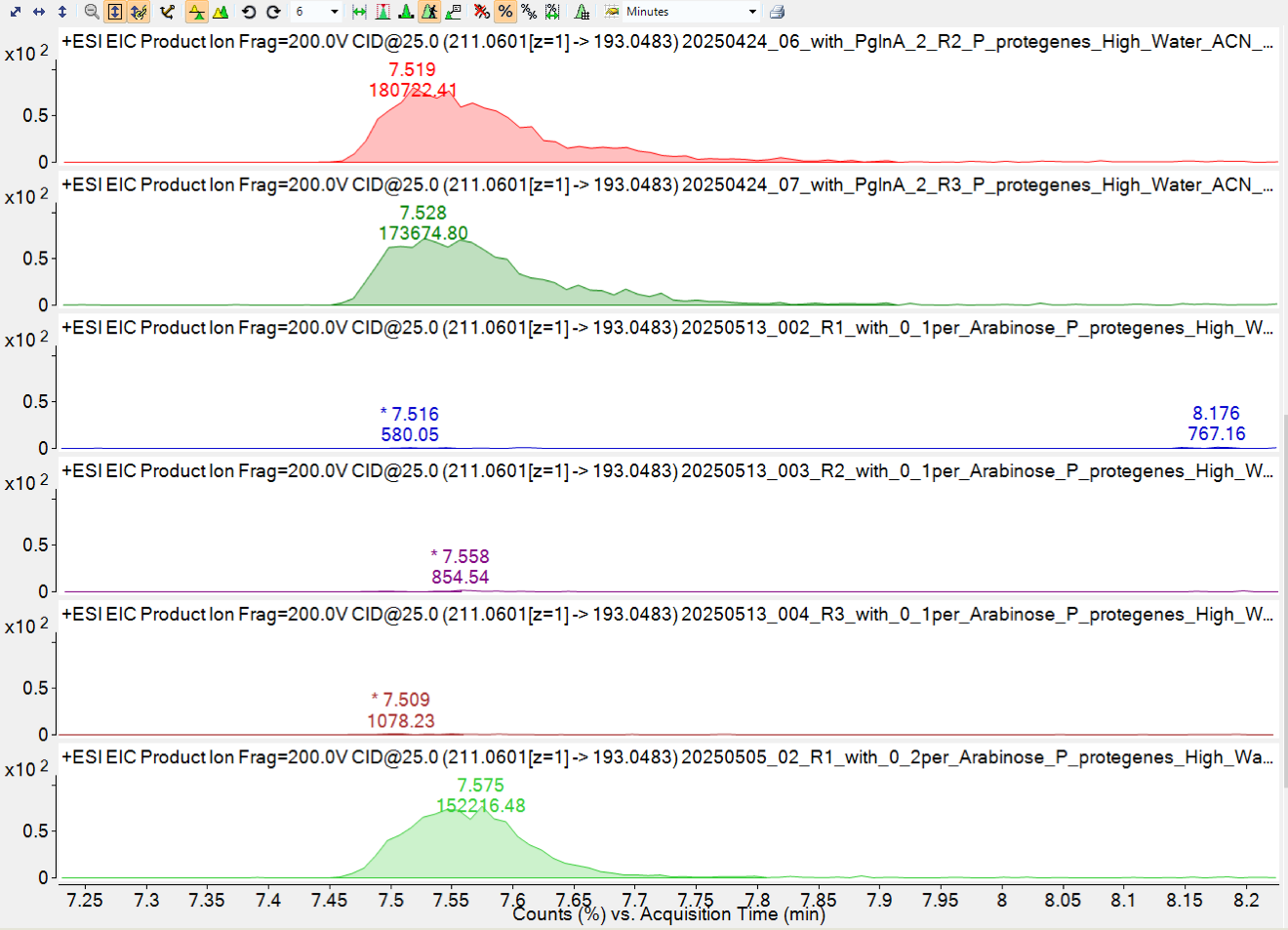


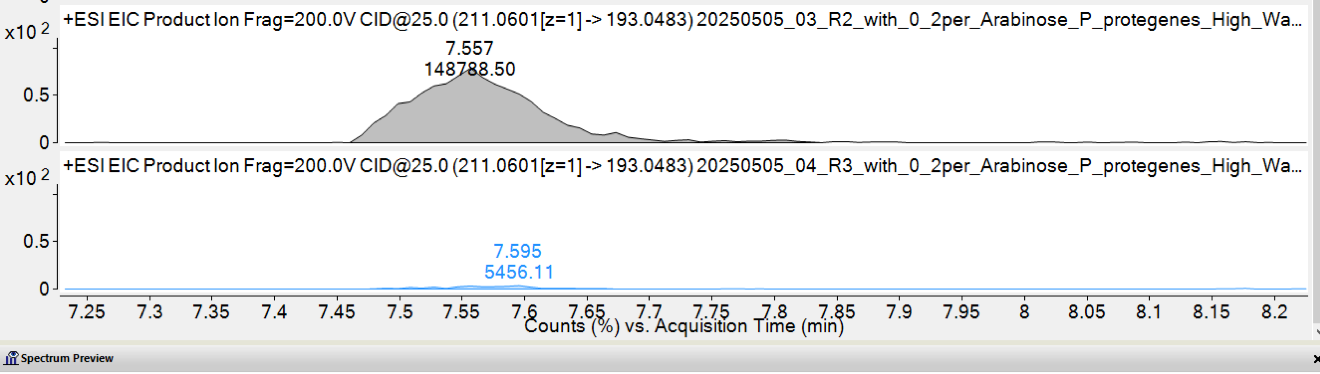


**Pyoluteorin (271.9876 m/z) day 03**

**
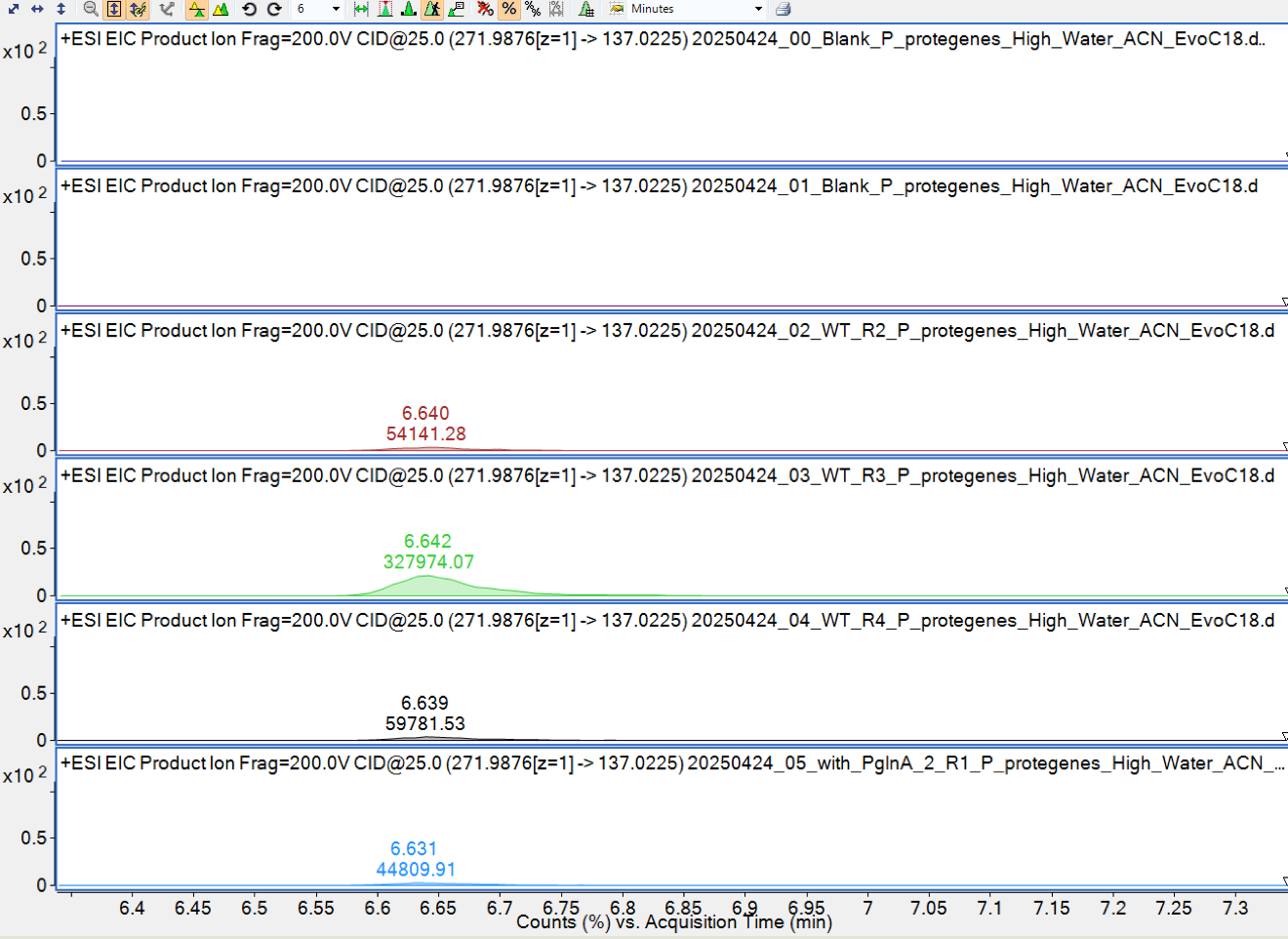
**

**
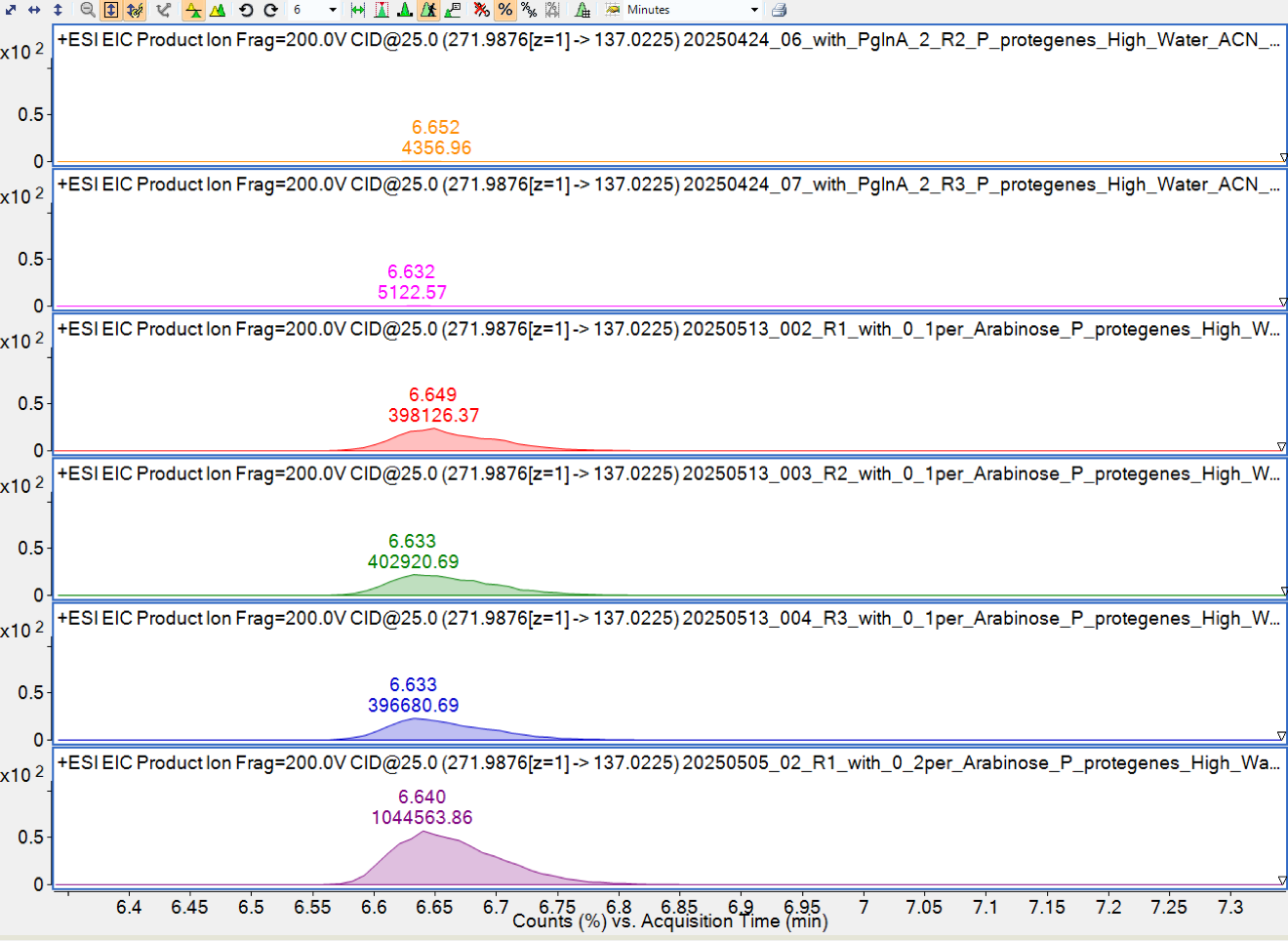
**

**
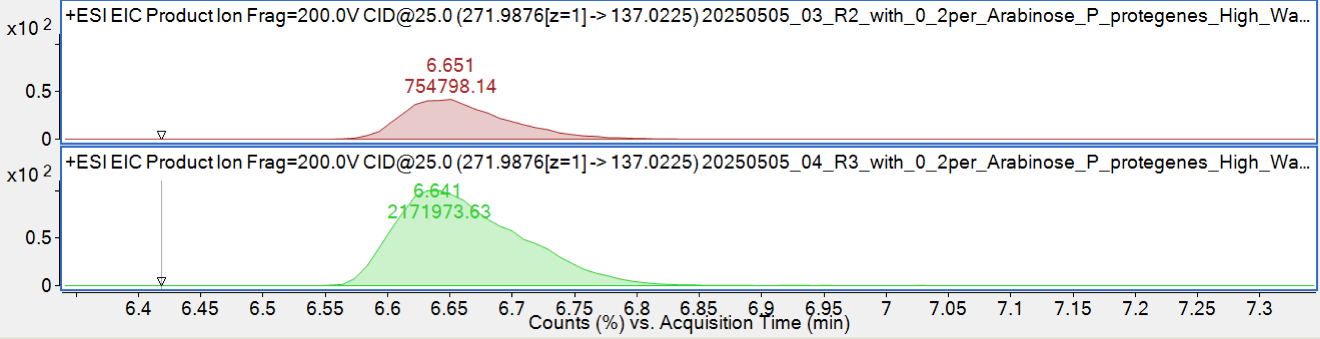
**

**Orfamide A (1295.8436 m/z) day 03**

**
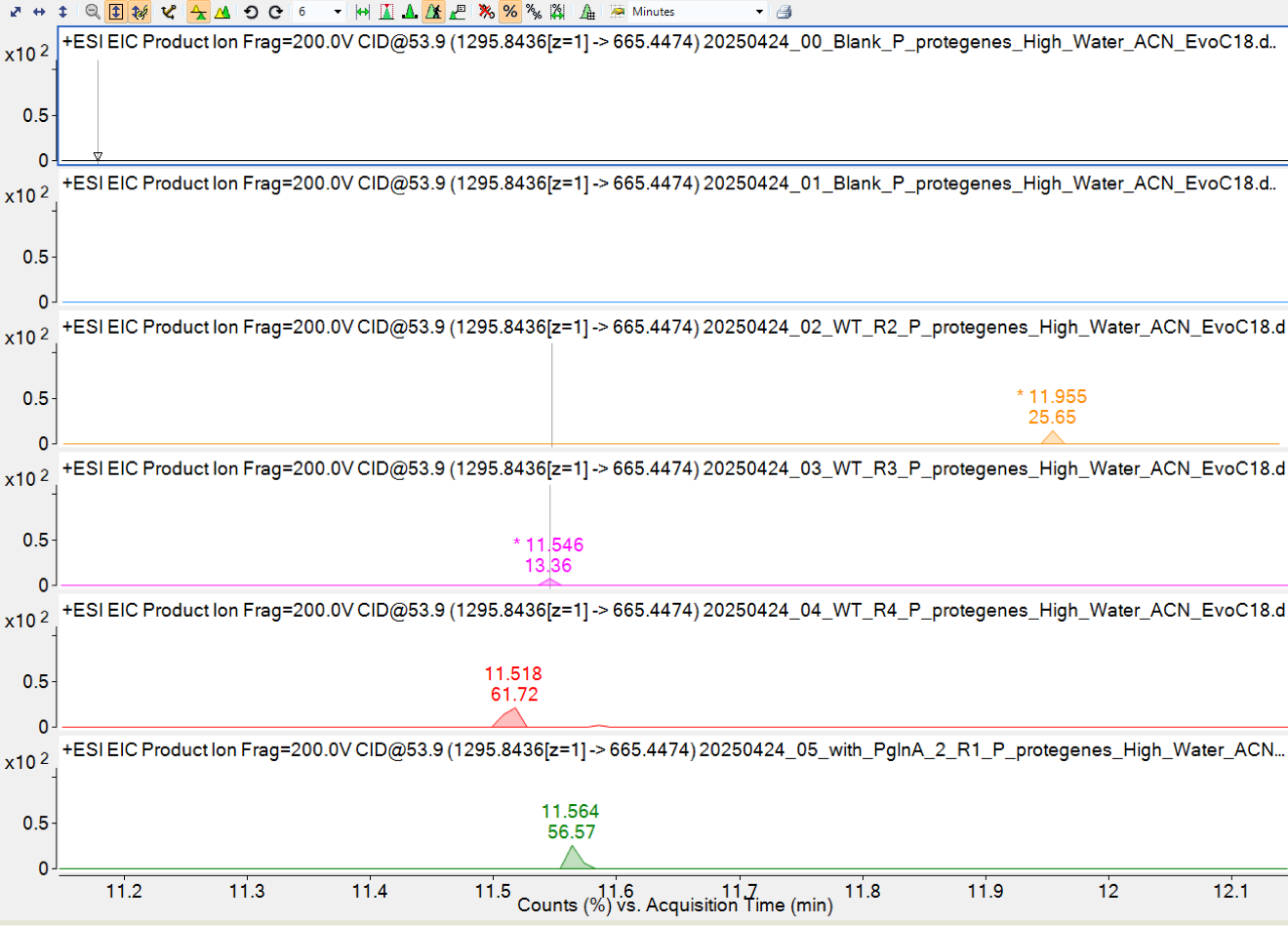
**

**
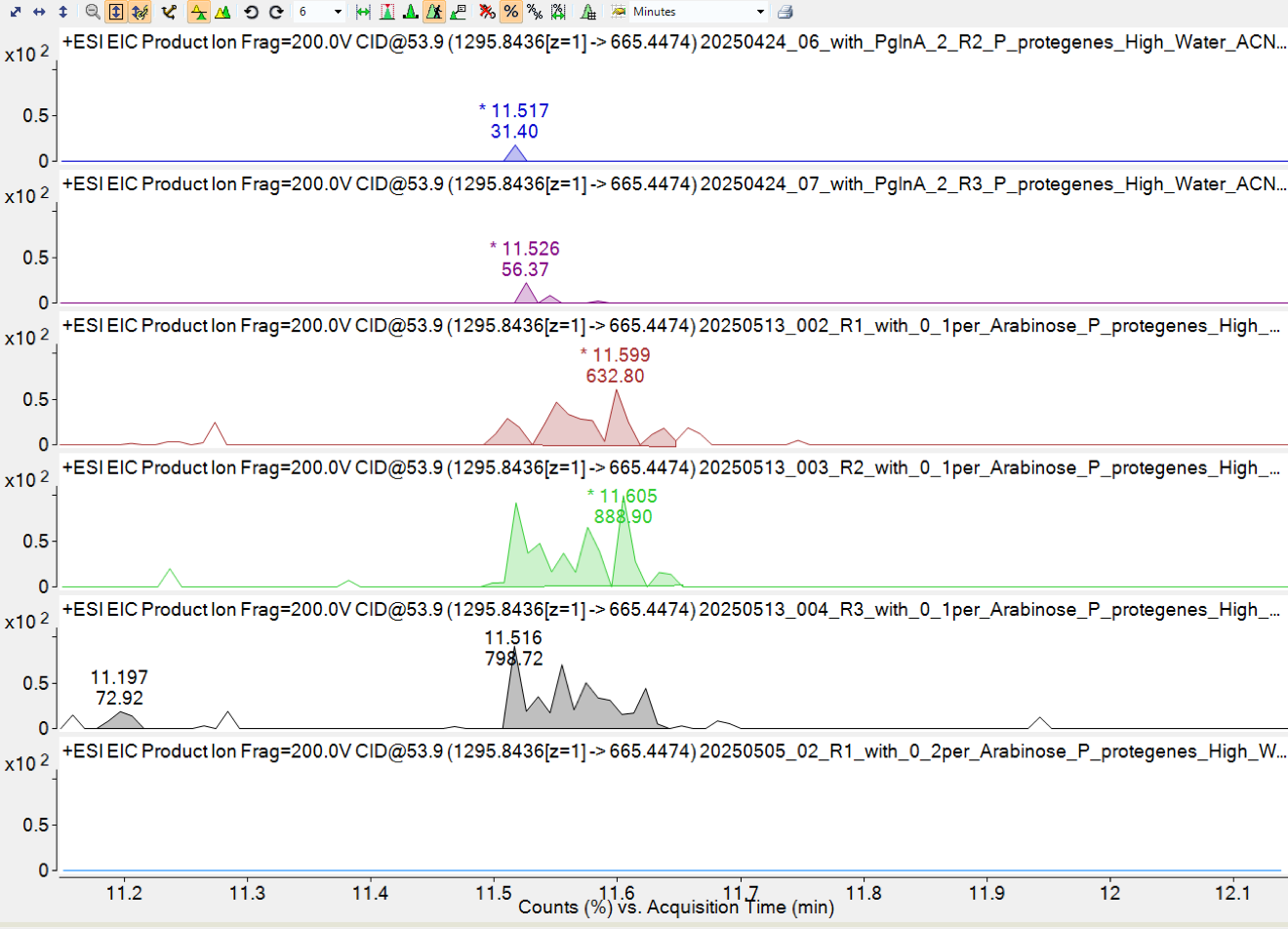
**

**
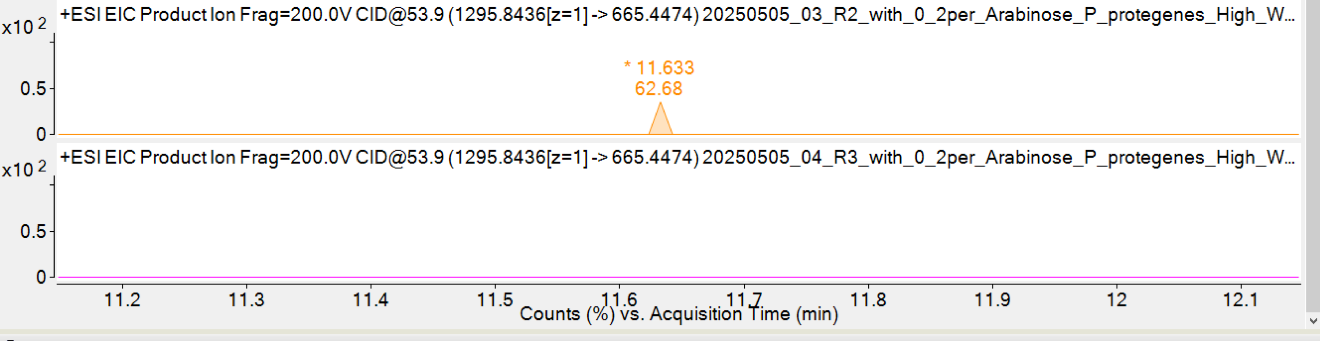
**

**--------------------------------**

**Figure S1: Targeted LC-MS/MS detection of secondary metabolites produced by *P. protegens*.** Shown are the extracted ion chromatograms for 2,4-DAPG (m/z 211.0601), pyoluteorin (m/z 271.9876), and orfamide A (m/z 1295.8436) in Day 3 samples.
