# Supplementary material for: Microgravity effects on secondary metabolism of plant-affecting bacteria
Source: Microbiol Spectr. 2026 Mar 30;14(5):e02239-25. doi: 10.1128/spectrum.02239-25 (PMC13141840; doi:10.1128/spectrum.02239-25)
Supplement: Fig. S2 — Shown are the extracted ion chromatograms for pyrroquinoline (m/z 256.1701) in Day 1 and Day 3 samples. [file spectrum.02239-25-s0003.docx]

**Burk_ambi**

**Quinoline Derivative 256.1701**

**
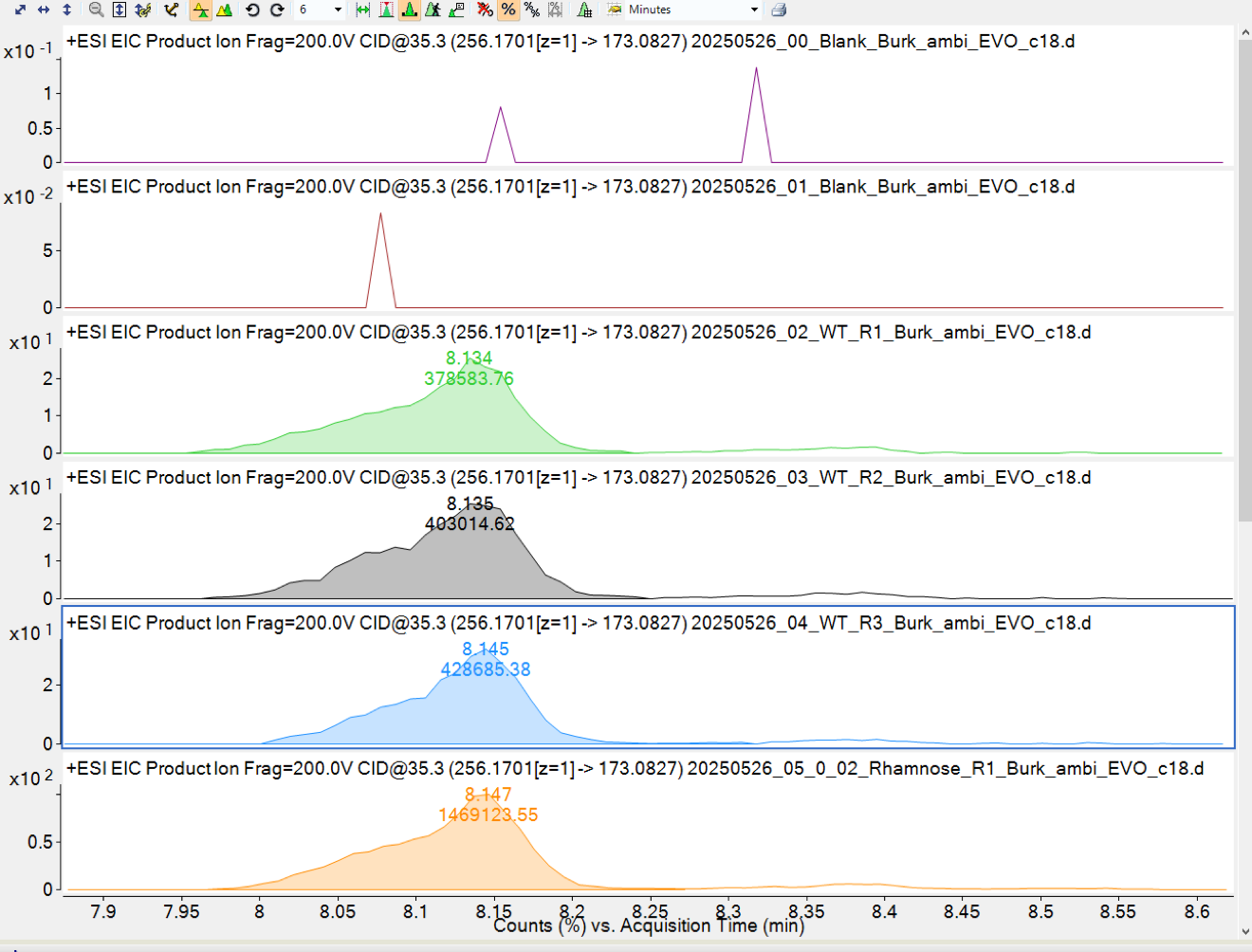
**

**
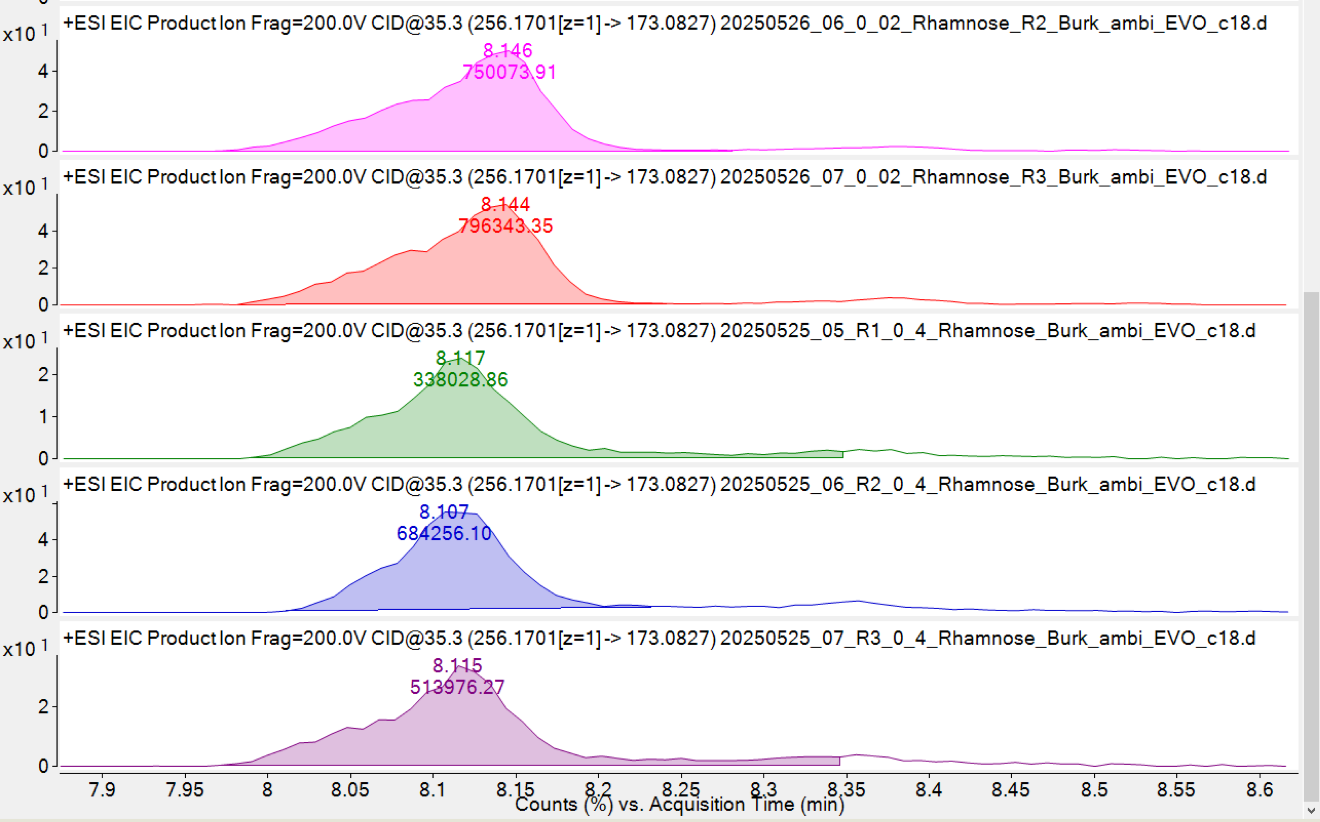
**

**FigureS2: Targeted LC-MS/MS detection of secondary metabolites produced by *B. ambifaria*.** Shown are the extracted ion chromatograms for pyrroquinoline (m/z 256.1701) in Day 1 and Day 3 samples
