# Supplementary material for: Microgravity effects on secondary metabolism of plant-affecting bacteria
Source: Microbiol Spectr. 2026 Mar 30;14(5):e02239-25. doi: 10.1128/spectrum.02239-25 (PMC13141840; doi:10.1128/spectrum.02239-25)
Supplement: Supplemental tables — Tables S6 to S9. [file spectrum.02239-25-s0009.docx]

| **Strain** | **Description** | **Source or reference** |
| --- | --- | --- |
| *Pseudomonas protegens Pf-5* | Plant promoting host strain | ATCC |
| *Burkholderia ambifaria AMMD* | Plant promoting host strain | ATCC |
| *E. coli* | DH5α-general cloning strain |  |
| *E.coli* | S-17- conjugation- Homologous recombination strain |  |
| PC0677 | pEX18Tc-P*_glnA_2_*, tet^R^ | This study |
| PC0679 | pEX18Tc-P_BAD_, tet^R^ | This study |
| PC0690 | pEX18Tp-*pqqBCDE*, trp^R^ | This study |
| PC0681 | pMP220- P*_rrA::lacZ_*, tet^R^ | This study |
| PC0682 | pMP220- P*_rpoD::lacZ_*, tet^R^ | This study |
| PC0683 | pMP220- P*_rpsA::lacZ_*, tet^R^ | This study |
| PC0684 | pMP220- P*_glnA_2::lacZ_*, tet^R^ | This study |

Table S6: List of Strains

| **Plasmid** | **Description** | **Source or reference** |
| --- | --- | --- |
| pGS0001 | pEX18Tc-P*_glnA_2_*, tet^R^ | This study |
| pGS0002 | pEX18Tc-P_BAD_, tet^R^ | This study |
| pGS0003 | pEX18Tp-*pqqBCDE*, trp^R^ | This study |
| pGS0004 | pMP220- P*_dnaG::lacZ_*, tet^R^ | This study |
| pGS0005 | pMP220- P*_rpsA::lacZ_*, tet^R^ | This study |
| pGS0006 | pMP220- P*_rrA::lacZ_*, tet^R^ | This study |
| pGS0007 | pMP220- P*_glnA_2::lacZ_*, tet^R^ | This study |

Table S7: List of Plasmids

| **Primers** | **Nucleotide sequence** | **Purpose** |
| --- | --- | --- |
| qPCRofaA-F | GCACGCGCTGTTCGCCTTCG | F primer for *ofaA* (RT-PCR and qPCR) |
| qPCRofaA-R | CCGGCCTGCCACTCTTGCACC | R primer for *ofaA* (RT-PCR and qPCR) |
| qPCRofaB-F: | GGCCTTCGACAGCCTGGAGC | F primer for *ofaB* (RT-PCR and qPCR) |
| qPCRofaB-R: | CAACTGCGCCAGCAGGTCGC | R primer for *ofaB* (RT-PCR and qPCR) |
| qPCRofaC-F: | GCTGGCGGTGCAGTACAGCG | F primer for *ofaC* (RT-PCR and qPCR) |
| qPCRofaC-R | CAGCAGGACTACGCCGGCAG | R primer for *ofaC* (RT-PCR and qPCR) |
| qPCRrpsAF | CGCGAGCGTATCTCCCTGGG | F primer of reference gene *rpsA* (*P. protegens* Pf-5) (RT-PCR and qPCR) |
| qPCRrpsAR | GCCAGGGTGATGATGGCGCC | R primer of reference gene *rpsA* (*P. protegens* Pf-5) (RT-PCR and qPCR) |
| PromrpoD-F | tctagaggatccccgggtacGATCAGCCCAAGGCGCCC | F primer to create P*_rpod_*::*lacZ* construct for β-gal assay |
| PromrpoD-R | ccaagctttcgcgagctcgaTCGGGAAGTGTATCTGCCGAAC | R primer to create P*_rpod_*::*lacZ* construct for β-gal assay |
| PromglnA_2-F | tctagaggatccccgggtacTTTATGTGCAGAAGGTTTTCGATGC | F primer to create P*_glnA_2_*::*lacZ* construct for β-gal assay |
| PromglnA_2-R | ccaagctttcgcgagctcgaTGGCTTCGGGCTTGGTGT | R primer to create P*_glnA_2_*::*lacZ* construct for β-gal assay |
| PromrpsA-F | tctagaggatccccgggtacCAAGAAGGATTGCAGGGGACC | F primer to create P*_rpsA_*::*lacZ* construct for β-gal assay |
| PromrpsA-R | ccaagctttcgcgagctcgaGGGCGAAGAATCCGCCCA | R primer to create P*_rpsA_*::*lacZ* construct for β-gal assay |
| PromrrA-F | tctagaggatccccgggtacGGCGAAGGCTCTGACAAG | F primer to create P*_rrA_*::*lacZ* construct for β-gal assay |
| PromrrA-R: | ccaagctttcgcgagctcgaCGAGAAACTCCTGTAACAGAAAG | R primer to create P*_rrA_*::*lacZ* construct for β-gal assay |
| CpCRpMP220F | GCCATTGGGATATATCAACGGTGG | F primer for cloning confirmation of β-gal assay constructs |
| CpCRpMP220R: | GGTGCCCCCCCTTCTCGAAC | R primer for cloning confirmation of β-gal assay constructs |
| 5pCRrpodGSP1: | GATACAGCGGTGAGTCGGTG | GSP1 primer of *rpoD* for 5’RACE- first round of amplification |
| 5pCRrpoDGSP2: | CGTGGTCCATGATGAAGCCG | GSP2 primer of *rpoD* for 5’RACE- second round of amplification |
| 5pCRrpoDGSP3 | CTTGTGGAAGGGGCAGCAGG | GSP3 primer of *rpoD* for 5’RACE- third round of amplification |
| 5pCRrpsAGSP1 | CAGAGAACCTGGCAGGAACG | GSP1 primer of *rpsA* for 5’RACE- first round of amplification |
| 5pCRrpsAGSP2 | GCGCTTGGCTTTTTCACGGG | GSP2 primer of *rpsA* for 5’RACE- second round of amplification |
| 5pCRrpsAGSP3 | GCGTGAACGGTTACCCAACG | GSP3 primer of *rpsA* for 5’RACE- third round of amplification |
| 5pCRrraAGSP1 | GCTTTCTCGGCCAGCATGTC | GSP1 primer of *rrA* for 5’RACE- first round of amplification |
| 5pCRrraAGSP2 | CCTTGACCAGCGAGTTGTCC | GSP2 primer of *rrA* for 5’RACE- Second round of amplification |
| 5pCRrraAGSP3 | CGCGACCACCGAAATTGCTG | GSP3 primer of *rrA* for 5’RACE- Third round of amplification |
| 5pCRgln_2GSP1 | GCTTTCAGGTGCTCTTCGGC | GSP1 primer of *glnA_2* for 5’RACE- first round of amplification |
| 5pCRgln_2GSP2 | GTTTCGTCGTCCGGCATCAG | GSP2 primer of *glnA_2* for 5’RACE- Second round of amplification |
| 5pCRgln_2GSP3 | GCATGGTCACGTGATGTTGAG | GSP3 primer of *glnA_2* for 5’RACE- Third round of amplification |
| AAP | GGCCACGCGTCGACTAGTACGGGIIGGGIIGGGII | abridged anchor primer for 5’Race (used with GSP1- First round PCR) |
| AUAP | GGCCACGCGTCGACTAGTAC | abridged universal anchor primer for 5’Race ( used with GSP2/3- Nested PCR) |
| ofaAupF | taaaacgacggccagtgccaCAGGCGCTCATCGAACTC | F primer to amplify upstream fragment of *ofaA* gene for creating P_BAD_-*ofaABC* |
| ofaAupR | ttagcgagaaCGGAGGGCTTTTGCGTTC | R primer to amplify upstream fragment of *ofaA* gene for creating P_BAD_-*ofaA*BC |
| PbadF | aagccctccgTTCTCGCTAACCAAACCGG | F primer to amplify P_BAD_ promoter fragment form pBADTrfp to create P_BAD_-*ofaA*BC |
| PbadR | ggcccatggcTATGGAGAAACAGTAGAGAGTTG | R primer to amplify P_BAD_ promoter fragment form pBADTrfp to create P_BAD_-*ofaABC* |
| ofaAdnF | tttctccataGCCATGGGCCGGGCACCC | F primer to amplify downstream fragment of *ofaA* gene for creating P_BAD_-*ofaABC* |
| ofaAdnR | acagctatgacatgattacgGGCGGTTCAGCACTGGCATGCCC | R primer to amplify downstream fragment of *ofaA* gene for creating P_BAD_-*ofaABC* |
| ConfPbadF | taaaacgacggccagtgccaCAGGCGCTCATCGAACTC | F primers for Gibson assembly confirmation of pGS001 and pGS002 (primer same as ofaAupF) |
| ConfPbadR | TCTGGTACTTGTCCAGCCAG | R primers for Gibson assembly confirmation in pGS002 and pGS002 |
| SeqPbadF | CTGCCAGGGCCTCGCCAAGG | F primer for sequencing confirmation of pGS001, pGS002 and *P. protegens* P_BAD_-*ofaABC* , P*glnA_2-ofaABC* after allelic replacement |
| SeqPbadR/Seq*glnA_2*R | GGCCGGAACGGCCCCTTGAG | R primer for sequencing confirmation of pGS001, pGS002 and *P. protegens* P_BAD_-*ofaABC* , P*glnA_2-ofaABC* after allelic replacement |
| ofaAup-gln-F | taaaacgacggccagtgccaCAGGCGCTCATCGAACTC | F primer to amplify upstream fragment of *ofaA* gene for creating P*glnA_2-ofaABC* |
| ofaAup-gln-R | tgcacataaaCGGAGGGCTTTTGCGTTC | R primer to amplify upstream fragment of *ofaA* gene for creating P*glnA_2-ofaABC* |
| PglnA_2_F | aagccctccgTTTATGTGCAGAAGGTTTTCGATGC | F primer to amplify P*_glnA_2_* promoter fragment from *P.protegens* Pf-5 to create P*glnA_2-ofaABC* |
| PglnA_2_R | ggcccatggcTGGCTTCGGGCTTGGTGTTG | R primer to amplify P*_glnA_2_* promoter fragment from *P.protegens* Pf-5 to create P*glnA_2-ofaABC* |
| ofaAdn-gln-F | cccgaagccaGCCATGGGCCGGGCACCC | F primer to amplify downstream fragment of *ofaA* gene for creating P*glnA_2-ofaABC* |
| ofaAdn-gln-R | acagctatgacatgattacgGGCGGTTCAGCACTGGCATGC | R primer to amplify downstream fragment of *ofaA* gene for creating P*glnA_2-ofaABC* (same as ofaAdnR) |
| qPCR pqqB-F | CGCAGATCGATCACGTGACGG | F primer for *pqqB* (RT-PCR and qPCR) |
| qPCR pqqB-R | CCGGGAATCGCGAACGGTTC | R primer for *pqqB* (RT-PCR and qPCR) |
| qPCR pqqC-F | CGGATCCTCGATCACGATGG | F primer for *pqq*C(RT-PCR and qPCR) |
| qPCR pqqC-R | GCCGCGCGAAATTGACGTAC | R primer for *pqqC* (RT-PCR and qPCR) |
| qPCR pqqD-F | CATGTACCGCCTGCAGTGG | F primer for *pqqD* (RT-PCR and qPCR) |
| qPCR pqqD-R | CTGAACAGCCGCTCGAGTTC | R primer for *pqqD* (RT-PCR and qPCR) |
| qPCR pqqE-F | GATCAAGGCGCACGGCTATC | F primer for *pqqE* (RT-PCR and qPCR) |
| qPCR pqqE-R | GCTGGTCGCGATTCAGCATC | R primer for *pqqE* (RT-PCR and qPCR) |
| qPCR rpsL-F | GTTCGCAAAGGCCGTCAGTC | F primer of reference gene *rpsL* (*B. ambifaria* AMMD) (RT-PCR and qPCR) |
| qPCR rpsL-R | CTGCAGGTTGTGGCCTTCAC | R primer of reference gene *rpsL* (*B. ambifaria* AMMD) (RT-PCR and qPCR) |
| PqqBCDE-F | tgaaattcagcaggatcacaATGCAGTGGACCACGCCGGC | F primer for *pqqBCDE* promoter fragment amplification form genomic DNA of *B. ambifaria* AMMD and cloning into pSCrhaB2 |
| PqqBCDE-R | ctcatccgccaaaacagccaTCAGCGCCCGACCGAATTAGCC | R primer for *pqqBCDE* promoter fragment amplification form genomic DNA of *B. ambifaria* AMMD and cloning into pSCrhaB2 |
| ConfPqqBCDEP1-F ConfPqqBCDEP1-R  ConfPqqBCDEP2-F  ConfPqqBCDEP2-R ConfPqqBCDEP3-F  ConfPqqBCDEP3-R ConfPqqBCDEP4-F  ConfPqqBCDEP4-R  ConfPqqBCDEP5-F  ConfPqqBCDEP5-R ConfPqqBCDEP6-F ConfPqqBCDEP6-R | GTGAACATCATCACGTTCATC  CGCACTGTCGCGGATCGCGC  GATCGTCGCCGTCGTGCTGTG  GCGCGAGATGGCCCATCTCG  CAGTGTGGCGACGCGGCGCAG  GCACGTACGCATCCTGGGCC  CTCCTGTATCCGGAAGGCATG  CTGCAGCGACAGCTGGATATG  GGATTCGACGCGCGAGCTG  CGCTGTCGTACCAGATTTCC  CAGCGACGCGTTCAACGCG  CACTTCTGAGTTCGGCATG | F and R primers for sequencing confirmation of pGS003 |

Table S8: List of Primers

| P*_ofaABC_* | CAGGCCATGAGCGTCGTTCACGCGTAGCCAATTTCCTACAGCCGATTCAGCTGATCAGCAGAGCTGCTGCAACAGCCCGGCGCGGTGCTGCCCGAGGTGGCCGACCGGGGCCGGAACTGTCCGGGTTTCGGGTGACATACAGGCCCTGGGGCCGATGCTTAAATCCGCGGACGCCGGTGCCCGGGCCCATCACATGAGAAGGATCTTATGACGCATTTTTCCGCCGCAGCCGTGGGCGCTTTCGTCGCGCCAGTCACCCCAGCCAGCCACTGCCC | [1] |
| --- | --- | --- |
| P*_rpoD_* | GATCAGCCCAAGGCGCCCTTCTCCAGCGCCCGCTTAATGAGCTATCCGAGGCCAGCCCAAAGCCACTGTCGGACTCAGCGCAACACCTTCAAATAGTCGAATACTGATTGAAACTAACGTCAGTGGACGTTCGGCAGATACACTTCCCGA**C** | This study |
| P*_rpsA_* | ACAAGAAGGATTGCAGGGGACCAGTCATAGTCCTGCGATCGTTCTTTTATATGAACGTA**A**CCCACATCGTCTGGGATGTGGCAGATGGGCGGATTCTTCGCCC | This study |
| P*_rrA_* | GGCGAAGGCTCTGACAAGGGCATTTCGCTCGATAGCGCTTGCGGCTAAGATATGACGCATTGCGCTGTTCTGGTCACATTCAGTTATTGAGTCGGCTTTCCGTTCCGCTGGCCTGGCTGCGAAAATACGGGGCTGGCGGGTTCGCTCATCAGGCCTTGCAGGTTAAACATCTGCGGTCTGGGCGCGTATCTATATAAGGCAGCACCGACACCT**T**CGATCGTGTTCTTTCTGTTACAGGAGTTTCTCG | This study |
| P*_glnA_2_* | TTTATGTGCAGAAGGTTTTCGATGCACCAAAACGGGTCATTTATTGGCAAAGACGCTACATTGCAGTGCGCCGAATGCCGGCATTCAACCCAATACGTCGCAGAAAGCGTGATTTTGGGGCAGAAAACCCATCTCGGGGCACTGGCATGCAATTTGCTCCCTTGTGAGGCAGGTTGCCATGGCAGAGTATTCGCG**C**CGGCATCACCCACATTCTAAGGGCACTCCAACACCAAGCCCGAAGCCA | This study |

Table S9: Promoter sequences

References
1. Muzlera, A.; Sobrero, P.; Agaras, B.; Valverde, C. Orfamide Production in Pseudomonas Protegens CHA0T Promotes Rhizospheric Colonization and Influences Assemblage of the Bacterial Community of Wheat Roots in Soil. *Rhizosphere* 2024, *30*.
